# A History of Corollary Discharge: Contributions of Mormyrid Weakly Electric Fish

**DOI:** 10.3389/fnint.2020.00042

**Published:** 2020-07-29

**Authors:** Matasaburo Fukutomi, Bruce A. Carlson

**Affiliations:** Department of Biology, Washington University in St. Louis, St. Louis, MO, United States

**Keywords:** efference copy, sensorimotor integration, electrosensory, electrolocation, communication, prediction, comparative physiology

## Abstract

Corollary discharge is an important brain function that allows animals to distinguish external from self-generated signals, which is critical to sensorimotor coordination. Since discovery of the concept of corollary discharge in 1950, neuroscientists have sought to elucidate underlying neural circuits and mechanisms. Here, we review a history of neurophysiological studies on corollary discharge and highlight significant contributions from studies using African mormyrid weakly electric fish. Mormyrid fish generate brief electric pulses to communicate with other fish and to sense their surroundings. In addition, mormyrids can passively locate weak, external electric signals. These three behaviors are mediated by different corollary discharge functions including inhibition, enhancement, and predictive “negative image” generation. Owing to several experimental advantages of mormyrids, investigations of these mechanisms have led to important general principles that have proven applicable to a wide diversity of animal species.

## Introduction

When we move our eyes to shift our gaze, a drastic change happens in our retinal image, but we still perceive a static visual scene. When we tickle ourselves, we hardly feel tickled. Thus, we must discriminate between environmental change-driven sensory input (exafference) and self-generated sensory input (reafference). These signals cannot be distinguished by sensory receptors. Instead, exafferent and reafferent stimuli are distinguished within the central nervous system using a corollary discharge or efference copy, which are internal copies of motor command signals that influence central sensory processing.

**FIGURE 1:**
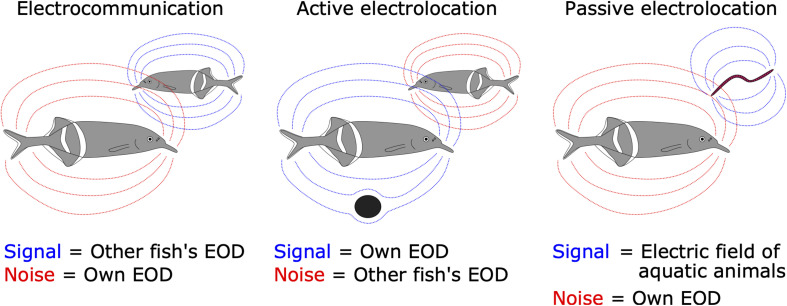
Signal and noise are different among the three electrosensory-mediated behaviors. For electrocommunication, EODs generated from neighboring fish (dashed blue lines) are signal while self-generated EODs (dashed red lines) are noise **(left)**. For active electrolocation, self-generated EODs (blue) are signal while EODs from other fish (red) are noise **(middle)**. For passive electrolocation, low-frequency weak electric fields generated from aquatic animals (e.g., worm) are signal (blue) while self-generated EODs (red) are noise **(right)**.

The concepts of corollary discharge and efference copy were proposed by Sperry (1950) and von Holst and Mittelstaedt (1950), respectively. Corollary discharge refers to any motor-related timing signal that influences sensorimotor processing. Efference copy has a narrower sense, referring to a subtractive signal for canceling predictable reafferent input. Since their discovery, neurobiologists have sought to identify mechanisms using diverse animal species. Studies of mormyrid weakly electric fish have contributed substantially to understanding the neural circuitry and mechanisms underlying corollary discharge. These fish generate stereotyped electric pulses termed electric organ discharges (EODs) from an electric organ located at the base of the tail. The EODs are used for two different behaviors. One is electrocommunication, in which fishes communicate their identities and behavioral states to each other (Hopkins, 1986a). The other is active electrolocation, in which fish can sense the environment by detecting distortions in their self-generated EOD (von der Emde, 1999). In addition, mormyrids can detect the external electric fields generated by all aquatic organisms, which is referred to as low-frequency passive electrolocation (Kalmijn, 1974). Importantly, self-generated EODs have different implications for these three behaviors (Figure 1). Reafferent inputs are noise for communication and passive electrolocation, whereas they are signal for active electrolocation. By contrast, exafferent input is noise for active electrolocation. The sensory processing related to these behaviors is performed by separate sensory pathways, each having a different type of sensory receptor (Bell, 1989; Perks and Sawtell, 2019). In these dedicated sensory pathways, corollary discharges differently modulate sensory processing to extract behaviorally relevant information (Bell, 1989; Perks and Sawtell, 2019).

Mormyrids have several advantages for studying neural mechanisms of corollary discharge. (1) In freely behaving fish, the motor command signal from spinal electromotor neurons is linked 1:1 with EOD output. (2) It is easy to record this motor command signal as a fictive EOD when the fish is immobilized and electrically silenced. (3) This recording of command signals is not invasive. (4) The recording site for motor commands is distant from the brain, which allows for simultaneous electrophysiological recording from the brain. (5) Stimuli that mimic reafferent EOD input can be delivered with arbitrary waveform and timing. Owing to these advantages, mormyrids have provided novel general insights into corollary discharge mechanisms in sensory processing.

There are numerous review papers describing mechanisms of corollary discharge in various sensory modalities and animals (e.g., Cullen, 2004; Poulet and Hedwig, 2007; Crapse and Sommer, 2008; Requarth and Sawtell, 2011; Schneider and Mooney, 2018; Straka et al., 2018). This review takes a historical perspective, emphasizing the critical contributions of research on mormyrids in advancing our understanding of corollary discharge mechanisms in sensory processing.

## Emerging Concepts of Corollary Discharge and Efference Copy

**FIGURE 2:**
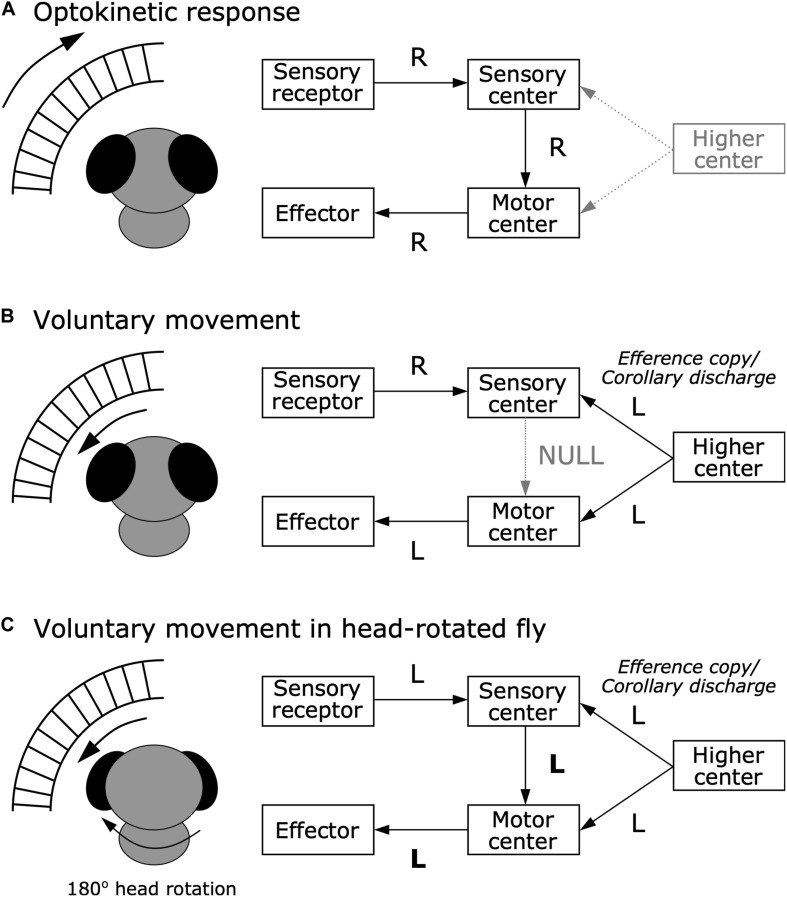
Efference copy hypothesis from the optokinetic response in blowfly. **(A)** Optokinetic response. When the external world moves rightward (R), sensory receptors tells the sensory center about this information. In turn, to stabilize the visual scene, the sensory center sends information about the rightward movement to the motor center, which executes the effector to move toward the right to maintain a stable visual image. **(B)** Voluntary movement in a normal fly. When the fly voluntarily moves leftward (L), rightward visual flow occurs. While the higher center provides the motor center with a command to move leftward, it also provides the sensory center with an efference copy or corollary discharge about the leftward movement command. This efference copy or corollary discharge signal can nullify the reafferent sensory signal, resulting in inhibition of the optokinetic response. **(C)** Voluntary movement in a head-rotated fly. When the 180° head-rotated fly moves leftward, leftward visual flow occurs. While the higher center provides the motor center with a command to move leftward, it also provides the sensory center with an efference copy or corollary discharge about the leftward movement command. However, since visual flow in the head-rotated fly is to the left rather than to the right, the efference copy or corollary discharge cannot nullify the reafferent signal, and instead amplifies it, resulting in continuous circling movements due to the optokinetic response.

In 1950, corollary discharge and efference copy were proposed independently by research groups in the United States and Germany. von Holst and Mittelstaedt (1950), who were German researchers, published a landmark paper titled *Das Reafferenzprinzip* (The Reafference Principle). In that paper, they discussed why stimuli that trigger reflexive behavior under stationary conditions do not evoke such reflexes when those stimuli are self-generated during voluntary behavior, referencing the optokinetic response of blowflies, postural reflex of fish, and bending reflex of millipedes. They proposed that an “efference copy” acts to subtract self-generated sensory input, or “reafference,” to distinguish from external sensory input, or “exafference.” For example, the optokinetic response is a reflex in which animals shift their gaze by moving their eyes or body in response to rapid changes in visual input (Figure 2A). This gaze shifting works to maintain visual field stability. A change in visual input also occurs when an animal voluntarily moves, but animals do not show optokinetic responses during voluntary movement (Figure 2B). von Holst and Mittelstaedt performed an experiment that rotated the fly’s head by 180 degrees about its longitudinal axis, which reversed its visual flow horizontally. They found that the head-rotated fly continuously circled after starting a voluntary movement in either direction (Figure 2C). This finding indicated that the optokinetic response was not simply inhibited during voluntary movements. Instead, they suggested the moving insect “expects” a specific visual stimulus due to its own movement, which is “neutralized” by an efference copy from the motor center. This could explain why the fly continued circling when the head rotation caused inverted visual flow, as the resulting reafferent sensory input would not be compensated, but instead enhanced by the efference copy.

Sperry, who was a neuropsychologist in the United States, first used the term “corollary discharge” (Sperry, 1950). Since “corollary” means something that is a direct or natural consequence of something else, Sperry used the term “corollary discharge” to refer to an internal signal that is the direct result of a motor command. Similar to the experiment by von Holst and Mittelstaedt, he focused on the optokinetic response of swellfish, *Sphaeroides spengleri.* He rotated one eyeball of the fish by 180 degrees, which also reversed its visual flow horizontally, while the other eyeball was covered with a foil blinder. He found a similar circling behavior in the eye-rotated fish. Further, he investigated the neural basis underlying this circling behavior by ablating vestibular organs or brain regions, including the optic tectum, forebrain, cerebellum, and/or inferior lobes. He found that ablation of the portion of the optic tectum that received input from the rotated eye abolished the circling behavior whereas ablation of the other regions had no effect. From these results, he predicted integration in the optic tectum between visual signals from the eye and corollary discharge signals of motor patterns that plays an important role in visual perception during voluntary movement.

Since emerging concurrently and independently, the terms corollary discharge and efference copy have often been used interchangeably. However, some previous reviews have described important differences between corollary discharge and efference copy (Crapse and Sommer, 2008; Straka et al., 2018). Efference copy, as its name suggests, is defined as a copy of an efferent motor command sent to the sensory pathway. The efference copy contains a subtractive signal for canceling predictable sensory input caused by an animal’s own behavior. In other words, if the reafferent input is regarded as a “positive image,” the efference copy is a “negative image” of the reafferent input. By contrast, corollary discharge has a more general meaning: a motor-related timing signal that influences sensorimotor processing. A corollary discharge can have many different effects including inhibition, facilitation, and modulation. Thus, the term corollary discharge encompasses efference copies and additional effects of motor-related signals on sensorimotor processing.

## Early Physiological Evidence of Corollary Discharges

Although there is no consensus as to who obtained the first physiological evidence of a corollary discharge, supporting data began to be published around the end of the 1960s.

### Possible Corollary Discharge Signals

The first evidence of a corollary discharge signal might have been found in goldfish (*Carassius auratus*), in relation to eye movement (Johnstone and Mark, 1969). Johnstone and Mark (1969) focused on the tectal commissure, which connects the left-right optic tecta, which directly receive inputs from retinal ganglion cells. They found that neurons in the tectal commissure showed two types of responses. One type of neuron exhibited regular discharge in the dark that was inhibited by applying light (Mark and Davidson, 1966). The other type had no spontaneous activity but exhibited high-frequency spikes in synchrony with flicking movements of the eyes (Johnstone and Mark, 1969). The authors interpreted the latter type of activity as a corollary discharge signal because: (1) stopping eye movements by paralyzing eye muscles did not affect this activity, suggesting it was not associated with sensory responses to eye movement; and (2) removal of the tectal commissure did not affect spontaneous eye movement, suggesting the commissure was not involved in the motor control of eye movement.

Another possible corollary discharge signal was found in the lateral-line system of the dogfish, *Scyliorhinus canicula*. The lateral-line hair cells monitor water flow surrounding the animal, which is drastically affected by self-generated sinuous movement during swimming. These hair cells are innervated by efferent fibers originating from the cerebellum (Hillman, 1969; Paul and Roberts, 1977). Roberts and Russell found that these efferent fibers were active when the fish was swimming, both spontaneously and when stimulated, whereas the fibers were silent when the fish was moved by the observer (Roberts and Russell, 1972). Because these efferent fibers provide inhibitory inputs to the hair cells, this system prevents the hair cells from being over-stimulated by self-generated movement.

### Suppression of Sensory Processing by Own Behavior

Around the same time, suppressive effects of behavior on sensory processing were found in various sensory modalities and taxa, suggesting a role for corollary discharges. To our knowledge, the first evidence for corollary discharge inhibition was found in the electrosensory system of a mormyrid, as we discuss in detail in a later section (Bennett and Steinbach, 1969). Here, we review motor-related suppression effects in other sensory modalities reported in the 1970s.

**FIGURE 3:**
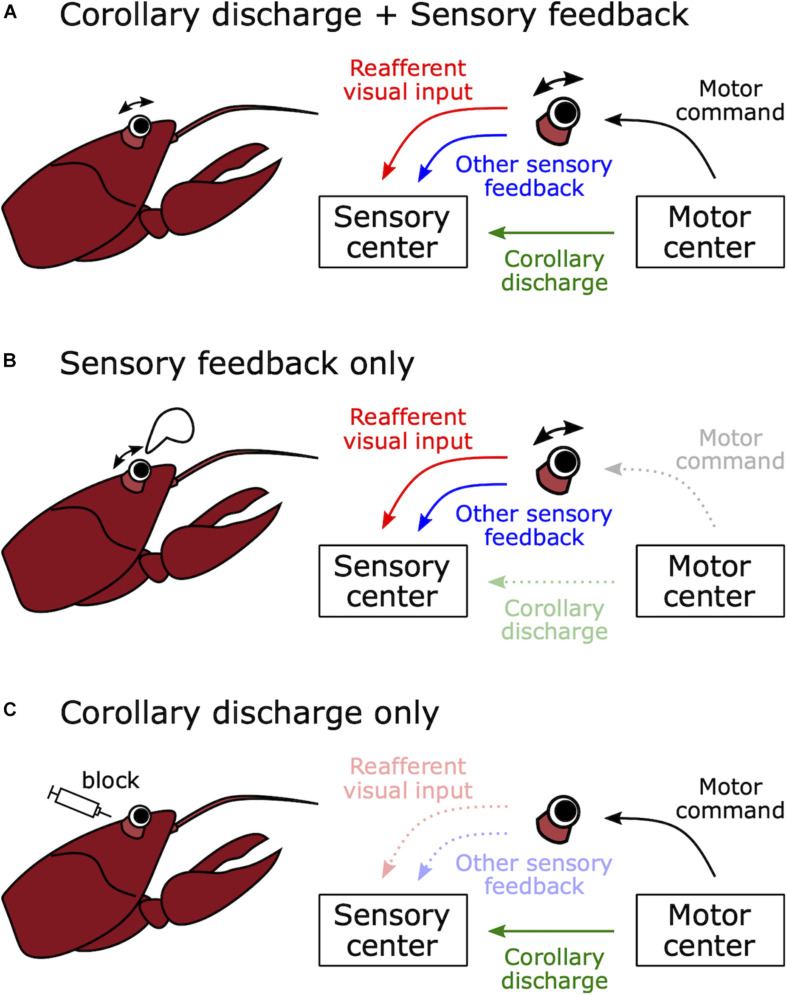
How to distinguish between sensory feedback and corollary discharge in mediating motor-related effects on sensory processing. **(A)** Natural voluntary behavior. When a crayfish moves its eye stalk, visual sensory processing may be modulated by corollary discharge signals from the motor control center or sensory feedback, for example from vestibular, proprioceptive, or coherent wide field visual inputs. **(B)** Passive movement of sensory organ. When the eye stalk is passively moved by an experimenter, there is no motor command and no corollary discharge signal. Thus, any effects of eye motion on the processing of visual stimuli must be due to sensory feedback. **(C)** Immobilized preparation. When the muscles involved in eye movement are curarized, there is no eye movement in response to a motor command and there is no reafferent visual input or sensory feedback. Thus, any changes in the processing of visual stimuli in response to motor commands must be due to a corollary discharge signal. In this case, even though eye movement is blocked, motor command signals from the motor center can be monitored as fictive movements.

Motor-related suppression in the visual system was found in a study of visual responses in optic fibers of crayfish (*Procambarus clarkii*) (Wiersma and Yamaguchi, 1967). Wiersma and Yamaguchi found optic fiber neurons that responded to moving visual stimuli but were unresponsive during active or passive (experimenter-induced) eye movements. Inhibition of visual responses during active eye movement might have been mediated by a corollary discharge (Figure 3A). However, inhibition during passive movement must have been mediated by sensory feedback about the eye movements (e.g., proprioception) because there was no internal motor command in this case (Figure 3B). Such feedback could also account for the inhibition observed during active eye movement (Figure 3A). As we will see, determining whether changes in sensory processing during behavior are due to sensory feedback or corollary discharge often requires experiments in which behavior is blocked such that central motor commands are decoupled from motor output (Figure 3C).

A similar kind of motor-related suppression was discovered in the superior colliculus of rhesus macaques, *Macaca mulatta* (Goldberg and Wurtz, 1972). Goldberg and Wurtz (1972) found that spontaneous activity of superior colliculus neurons was suppressed by eye movement in total darkness. In this case, because this suppression effect slightly preceded the eye movement, it was most likely due to a corollary discharge rather than sensory feedback.

Motor-related suppression was also found in the auditory system of the gray bat, *Myotis grisescens*. To navigate in a dark environment, bats emit ultrasound pulses and utilize information from the echo. Suga and Schlegel (1972) recorded auditory responses from the auditory nerve and the lateral lemniscus, which is a tract of axons relaying auditory information from the cochlear nuclei to the inferior colliculus. They found that the evoked potential response of the lateral lemniscus to self-vocalized sound was weaker than playback of the same sound, even though playback intensity was the same as the vocalization. Because auditory nerve responses were equivalent between these two sounds, this attenuation must have occurred between the auditory nerve and the inferior colliculus. In turn, Suga and Shimozawa (1974) explored where the attenuation actually occurs and identified it in the nucleus of the lateral lemniscus. This suppression mechanism likely acts to prevent habituation in response to the loud pulse and maintain sensitivity to the subsequent echo. However, these studies could not determine whether a corollary discharge or sensory feedback resulting from vocalization mediated this attenuation (see Figure 3).

Motor-related suppression was also found in the mechanosensory system. Crickets have organs called cerci at the rear of the abdomen, which have mechanosensory hairs that detect air flow (reviewed in Casas and Dangles, 2010). The cerci detect the rapid air flow that accompanies the approach of a predator, which triggers an escape response. However, the cerci also respond to air flow caused by self-locomotion. Murphey and Palka (1974) found that second-order neurons were less responsive to mechanosensory stimuli during walking compared to resting. Further, they made intracellular recordings from an identified neuron (medial giant interneuron; MGI) in a restrained preparation while monitoring extracellular neural activity from the ipsilateral middle leg nerve. The MGI showed an inhibitory postsynaptic potential during spontaneous burst firing of the leg nerve. Note that, by eliminating actual movement and monitoring fictive movement from the leg nerve, these experiments succeeded in eliminating sensory feedback as a possible cue, thereby demonstrating that this inhibition was mediated by a corollary discharge (see Figure 3C).

### Corollary Discharge Circuits Mediating Behaviors

How does corollary discharge govern an animal’s natural behavior? Compared to vertebrates, invertebrates have a small number of identifiable neurons in the central nervous system, which attracts neurobiologists who seek to understand neural circuits underlying behavior at a cellular level. In the mid-1970s, neural circuits involving corollary discharges were identified in sea slugs and crayfish.

Like swimming and walking, feeding behavior consists of rhythmic movements. Davis et al. (1973) examined neural circuits governing rhythmic feeding behavior in the sea slug *Pleurobranchaea californica*. While they identified motor neurons in the buccal ganglia that produced rhythmic oscillations during feeding, they also found neurons that send a corollary discharge associated with these oscillations to the brain (Davis et al., 1973; Siegler et al., 1974). Moreover, Gillette and Davis identified a command neuron in the brain, termed metacerebral giant (MCG), that triggers the rhythmic feeding behavior (Gillette and Davis, 1977). The MCG receives corollary discharge inhibition from the buccal ganglion, as well as tactile mechanosensory and chemosensory inputs related to food from the mouth. The corollary discharge feedback associated with feeding oscillations serves to amplify the rhythmic excitatory drive for feeding (Gillette and Davis, 1977). These studies were the first to demonstrate that corollary discharge governs rhythmic motor output during behavior.

A corollary discharge was also found to mediate behavioral choice in *Pleurobranchaea californica*. The sea slug normally exhibits a withdrawal response to vigorous tactile stimulation of the oral veil, whereas it starts rhythmic feeding behavior in response to chemical stimulation from food. When both stimuli are present, the sea slug shows feeding behavior, but does not exhibit the withdrawal response (Davis et al., 1973, 1977). Kovac and Davis (1977) identified one pair of corollary discharge interneurons from the feeding circuit that suppressed the activities of withdrawal motor neurons in response to tactile stimulation. Later, Kovac and Davis (1980) revealed this corollary discharge interneuron directly inhibited the withdrawal command neuron. This inhibition therefore acts to suppress withdrawal in response to self-generated tactile stimulation during feeding. More generally, these findings described a cellular basis for behavioral choice governed by corollary discharge inhibition.

The crayfish *Procambarus clarkii* exhibits a rapid escape response to mechanosensory stimuli. The neural circuit underlying this tail-flip escape behavior has been well characterized (Wiersma, 1947; Edwards et al., 1999): mechanosensory stimulation to the caudal body activates lateral giant (LG) fibers to elicit upward-jumping escape while stimulation to the rostral body activates medial giant (MG) fibers to elicit backward escape. These giant fibers receive mechanosensory inputs via second-order sensory interneurons. Strong mechanosensory stimulation is generated from spontaneous movements, including the tail-flip, which would strongly activate many mechanosensory afferents. Krasne and Bryan (1973) examined how crayfish discriminate such self-generated stimuli from external mechanosensory stimuli. They found that a corollary discharge signal from the tail-flip motor circuit provides presynaptic inhibition to the synapse between the mechanosensory afferents and the interneurons, which can protect the animal from maladaptive habituation and prevent repeated activation of the escape circuit in response to the animal’s own movement. Thus, this study also delineated a cellular-level circuit involving a corollary discharge that governs behavior.

## Corollary Discharge Inhibition for Communication

Many animals communicate with conspecifics by exchanging signals such as sounds. In communication, each sender is also a receiver of others’ signals. The problem here is that the sender receives an intense stimulus from their own signal production, which represents a source of noise in processing other individuals’ signals and may lead to desensitization through habituation (Figure 1). How does the central nervous system address this problem?

### Electrocommunication in Mormyrid Weakly Electric Fish

Understanding corollary discharge mechanisms underlying communication began with the study of a mormyrid fish, *Gnathonemus petersii*. As mentioned in the introduction, mormyrid fish generate EODs from an electric organ, and distinct sensory pathways govern three different electrosensory behaviors: electrocommunication, active electrolocation, and low-frequency passive electrolocation. Before the discovery of a corollary discharge in mormyrids, it was thought that the neural pathway that mediates communication derives from a relatively large type of electroreceptor called the Knollenorgan (KO) (Bennett, 1965). The reasons why the KO was thought to mediate communication are (1) sensitivity to high-frequency signals characteristic of EODs, (2) a fixed-latency spike of primary afferents in response to EODs that is largely amplitude invariant, and (3) the greatest sensitivity among the three types of electroreceptors. Together, these properties suggested this receptor is specialized to detect electric signals from other fish.

**FIGURE 4:**
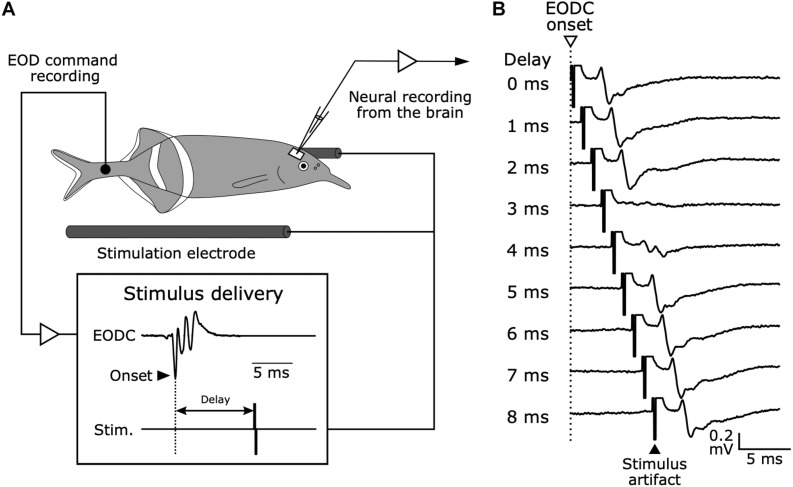
Electrophysiology in mormyrid fish brains while monitoring EOD command signals and delivering time-locked stimuli. **(A)** Experimental setup. Although the fish is curarized to eliminate movement and silence EOD production, EOD commands (EODC) from spinal electromotor neurons can be recorded as fictive EODs using an extracellular electrode placed next to the tail. Electrosensory stimuli can be delivered at fixed delays relative to the EODC onset. This system allows for the examination of corollary discharge effects on electrosensory neurons in the brain and to separate corollary discharge effects from the effects of sensory feedback. Modified from Bell (1981). **(B)** Evoked potentials from the exterolateral nucleus anterior (ELa) in response to stimuli at varying delays following EOD command onset (0–8 ms) in *G. petersii.*

Bennett and Steinbach published the idea that electrosensory processing needs information about when an EOD is produced to extract behaviorally relevant information (Bennett and Steinbach, 1969). They tested whether neural signals related to EOD production were observed in sensory areas across the brain. They used a preparation of curarized (muscle-inactivated) fish, in which the EOD is silenced but fish continue to produce fictive EODs from spinal electromotor neurons (Figure 4A). This preparation has the powerful advantage that silencing the EOD can isolate the effects of a corollary discharge on sensory processing by eliminating sensory feedback (see Figure 3C). They found that the cerebellum and the electrosensory lateral line lobe, in which the electrosensory afferents terminate, both received corollary discharges reflecting the timing of EOD production. In addition, they showed that sensory responses of the exterolateral nucleus in the midbrain torus semicircularis disappeared when electrosensory stimuli were delivered within a narrow window of time shortly after the fictive EOD (Figure 4B). Later, it was shown that the exterolateral nucleus appeared to receive electrosensory inputs from KO afferents via the hindbrain nucleus of the electrosensory lateral line lobe (nELL) (Enger et al., 1976a, b). Taken together, it was shown that the KO pathway can efficiently extract communication signals from other fish by internally canceling responses to self-generated signals using a corollary discharge.

**FIGURE 5:**
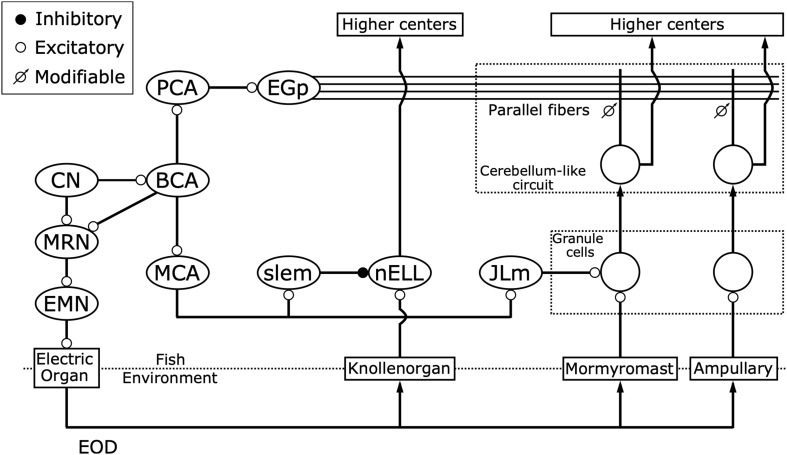
Corollary discharge pathways interact with three distinct electrosensory pathways. While the command nucleus (CN) drives the electric organ to generate each EOD via the medullary relay nucleus (MRN) and spinal electromotor neurons (EMN), it also provides a corollary discharge via the bulbar command-associated nucleus (BCA). Knollenorgans, which are dedicated to communication, send their primary afferents to the nucleus of the electrosensory lateral line lobe (nELL), which receives corollary discharge inhibition from the BCA via the mesencephalic command-associated nucleus (MCA) and the sublemniscal nucleus (slem). Mormyromast and ampullary receptors, which are dedicated to active electrolocation and passive electrolocation, respectively, send their afferents to granule cells of the electrosensory lateral line lobe (ELL). Only granule cells that are innervated by mormyromast afferents receive corollary discharge enhancement from the BCA via the MCA and the medial juxtalobar nucleus (JLm). Both granule cells send their outputs to medium ganglion (MG) cells, which also receive inputs onto their apical dendrites from parallel fibers that come from the eminentia granularis posterior (EGp), forming cerebellum-like circuits. The EGp provides corollary discharge inputs to the MG cells via the BCA and the paratrigeminal command-associated nucleus (PCA). In these cerebellum-like circuits, a “negative image” of expected reafferent input is made through anti-Hebbian spike-timing-dependent plasticity at the synapses between parallel fibers and the apical dendrites of MG cells. Modified from Bell (1989); Perks and Sawtell (2019).

**FIGURE 6:**
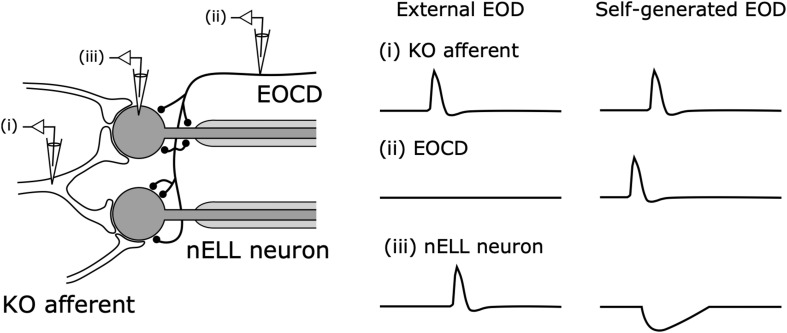
Corollary discharge inhibition in the nucleus of the nELL. Primary Knollenorgan (KO) afferents form large excitatory synapses onto the soma of adendritic nELL neurons. The electric organ corollary discharge (EOCD) from the sublemniscal nucleus (slem) also provides inhibitory inputs onto the soma and initial segment of nELL neurons. In response to an external EOD, (i) KO afferents and (iii) nELL neurons produce spikes whereas (iii) the EOCD is not activated. In response to self-generated EODs, (ii) slem neurons produce a spike preceding (i) the KO afferent spike, resulting in: (iii) nELL neurons showing an inhibitory postsynaptic potential that blocks the spiking response to afferent input. Modified from Bell and Grant (1989); Carlson (2009a).

The question that followed was what neural pathways mediate this corollary discharge inhibition. Zipser and Bennett (1976) found that the corollary discharge inhibition occurred in the nELL, the first sensory center of the KO pathway (Zipser and Bennett, 1976). In turn, using horseradish peroxidase tracing, Bell et al. (1981) revealed that, in addition to input from KO primary afferents, the nELL also received inputs from a small group of cells, later named the sublemniscal nucleus (slem) (Mugnaini and Maler, 1987). Furthermore, Bell et al. (1983) described a corollary discharge pathway from the EOD command nucleus (CN) to the slem through the bulbar command-associated nucleus (BCA) and mesencephalic command-associated nucleus (MCA) (Figure 5). The input from the slem appeared to be GABAergic based on immunocytochemistry (Denizot et al., 1987; Mugnaini and Maler, 1987). Since the neural activity in the CN corresponds 1:1 to EOD production, this pathway was strongly suggested to provide corollary discharge inhibition to the nELL. Indeed, Bell and Grant performed intracellular recording from the nELL, including nELL neurons, KO primary afferents, and inhibitory inputs from slem, and revealed the neural circuit of corollary discharge inhibition physiologically (Figure 6; Bell and Grant, 1989).

These studies elucidated a corollary discharge circuit and mechanism underlying communication for the first time. In addition, they suggested that, since the corollary discharge inhibition of the nELL can preserve the temporal information of communication signals from other fish, the downstream pathway should analyze temporal features of the signal. Indeed, future studies demonstrated that the ELa extracts information about temporal features of the EOD waveform that reflect the identity of signaling fish (Friedman and Hopkins, 1998; Lyons-Warren et al., 2013), and that the posterior exterolateral nucleus (ELp), to which ELa sends its only output, extracts temporal patterns of inter-pulse intervals that reflect the behavioral state of signaling fish (Carlson, 2009b; Baker et al., 2016). Owing partly to this corollary discharge inhibition, mormyrid fishes provided a unique opportunity to study how the nervous system decodes temporal signals during communication (Xu-Friedman and Hopkins, 1999; Baker et al., 2013).

### Acoustic Communication in Primates

Similar to electric communication in mormyrids, corollary discharge inhibition may mediate acoustic communication in primates, including humans. Like prior research on bats (Suga and Schlegel, 1972; Suga and Shimozawa, 1974), Müller-Preuss and Ploog (1981) compared sensory responses of auditory cortex to playback calls and self-vocalized calls in squirrel monkeys (*Saimiri scireus*) and found that the auditory response was absent during vocalization. A similar effect was subsequently found in human cortex (Creutzfeldt et al., 1989).

The anatomy of a possible corollary discharge pathway underlying communication in primates remains controversial today. One possible source seems to be the prefrontal cortex, because (1) there are reciprocal connections between the auditory cortex and prefrontal cortex (Müller-Preuss et al., 1980) and (2) electrical stimulation of the prefrontal cortex can suppress responsiveness in the auditory cortex (Alexander et al., 1976). However, the electrical stimulation in the latter case did not necessarily reproduce the same activity that results from real vocalization. In addition, it remains unclear whether a corollary discharge directly inhibits the auditory cortex. Vocalization-induced suppression was not observed in the inferior colliculus of monkeys (Pieper and Jürgens, 2003), in contrast to findings in bats (Suga and Shimozawa, 1974), and it is not known whether thalamic or thalamocortical mechanisms participate in the vocalization-induced suppression observed in auditory cortex. A recent review paper provides further discussion of corollary discharge mechanisms in the auditory system of primates (Eliades and Wang, 2019).

### Acoustic Communication in Crickets

Male crickets produce song by rhythmically rubbing the forewings together to attract female crickets. The song is quite loud at the source (over 100 dB SPL) so that distant conspecifics can hear it (Nocke, 1972). This means that a singing cricket is fully exposed to the loud self-generated sound, which strongly stimulates the auditory tympanal organs located on the front legs. Early behavioral evidence showed that crickets can respond to external sound during singing (Heiligenberg, 1969), suggesting the existence of a corollary discharge. A series of later studies by Poulet and Hedwig clearly delineated the neural circuit underlying corollary discharge inhibition of the auditory pathway at the level of identified cells in singing field crickets, *Gryllus bimaculatus* (Poulet and Hedwig, 2002, 2003a,b, 2006).

Compared to mormyrids, insects have a distinct experimental advantage: individual neurons can be identified. However, there were two challenges to be worked out. (1) It is rare for crickets to sing during electrophysiological experiments. (2) The forewing movement during singing is very fast, and a method to detect and quantify this movement was needed. Hedwig (2000a, b) addressed these problems. (1) They found that injection of acetylcholine and cholinergic agonists into the brain can reliably trigger singing through activation of a command neuron (Wenzel and Hedwig, 1999; Hedwig, 2000b). (2) Hedwig developed an opto-electronic system to record wing movement at a 5 kHz sampling rate (Hedwig, 2000a).

Using these methods and intracellular recording, Poulet and Hedwig recorded from auditory neurons in the prothoracic ganglion, where the auditory afferents terminate, during singing (Poulet and Hedwig, 2002, 2003a,b). First, they showed that the auditory neurons responded with bursts of spikes to a cricket’s own singing sounds. However, the resulting spike rate was lower than the response to 100 dB SPL sound pulses at rest, suggesting inhibition of the auditory system during singing. Second, they prevented sound production while still allowing for wing movement by removing one forewing and directly showed inhibitory postsynaptic potentials (IPSPs) in phase with wing movement in the auditory neurons. Third, they isolated corollary discharge effects from sensory feedback (see Figure 3C) by cutting motor and sensory nerves except for auditory nerves and showed that the IPSPs continued to occur in phase with fictive singing as recorded from the wing motor nerve root. These results demonstrated that a singing-related corollary discharge inhibits the auditory neurons’ responses and prevents self-induced desensitization.

In a follow-up study, Poulet and Hedwig identified a corollary discharge interneuron (CDI) responsible for this inhibition (Poulet and Hedwig, 2006). The CDI has its dendrites in the mesothoracic ganglion, where the motor neurons innervating the wing muscles are found. However, the CDI is not involved in generating song. With dual intracellular recordings from the CDI and auditory neurons, they showed that activation of CDI induced suppression of auditory neurons while inactivation of CDI removed the effects of inhibition on auditory neurons during singing. This suggested that the CDI is necessary and sufficient to provide corollary discharge inhibition. These studies described for the first time the cellular basis for corollary discharge inhibition underlying acoustic communication.

Around the same time, Weeg et al. (2005) recorded from efferent neurons that innervate the inner ear and lateral line of a sound-producing fish, the plainfin midshipman (*Porichthys notatus*). Most of these neurons showed an increase in activity that was time-locked to the fine temporal structure of evoked fictive vocalizations. In addition, the activity of efferents projecting to the inner ear was suppressed just after the end of each fictive vocalization. These findings suggest that a corollary discharge of vocalizations acts to modulate auditory sensitivity to self-generated sounds and maintain sensitivity to external sounds. This is similar to the findings in crickets, and suggests that similar mechanisms may be operating across vocalizing invertebrate and vertebrate species.

## Corollary Discharge Enhancement for Active Sensing

Active sensing is acquiring sensory inputs through overt sampling behaviors, which requires sensorimotor interactions in a different manner from communication. In the context of communication, corollary discharges act to inhibit sensory responses. In the context of active sensing, however, corollary discharges can act to enhance sensory processing. Only two study systems, mormyrids and bats, have been used to study corollary discharges or motor-related enhancement during active sensing. In both systems, motor-related signals serve to gate sensory responses to self-generated behavioral outputs through enhancement.

### Active Electrolocation in Mormyrid Fish

Weakly electric fishes use EODs to sense their environment. Unlike sounds, electric signals do not propagate as traveling waves but exist as localized electrostatic fields (Hopkins, 1986b). This means that reflected echoes, which bats use during echolocation (Griffin, 1958), are not relevant to electric fish. Instead, objects near the fish alter the EOD-evoked current flow across receptors and project an “electrical image” onto the skin (reviewed in von der Emde and Bell, 2003). Objects with conductivity greater than the surrounding water project an electrical “brightspot” onto the skin, whereas objects with conductivity lower than the surrounding water project an electrical “darkspot” onto the skin.

Before discovering a corollary discharge underlying active sensing, researchers thought the mormyromast pathway has a major role in active electrolocation because the mormyromast receptors: (1) have high sensitivity to high-frequency signals characteristic of EODs; (2) show intensity dependencies in spike latency and number of spikes produced by the afferents, which could encode stimulus amplitude related to the size and location of objects; (3) are less sensitive than Knollenorgans (Bennett, 1965). In addition to these specialized features of mormyromasts, active electrolocation requires information regarding when the EODs were produced (Bennett and Steinbach, 1969).

Zipser and Bennett (1976) made intracellular recordings from neurons in the electrosensory lateral line lobe (ELL) that received inputs from the mormyromast afferents and found that neural responses were facilitated within a narrow time window (6–11 ms) with respect to EOD command onset. This suggested that a corollary discharge gated self-generated responses. In contrast to the nELL in the Knollenorgan pathway, the ELL cortex has a laminar structure that includes various types of neurons (Maler, 1973). However, Zipser and Bennett did not determine what cell types were responsible for corollary discharge enhancement of electrosensory responses. With intracellular recording and morphological analysis, Bell et al. (1989) later identified granule cells as the convergent site of corollary discharge inputs and mormyromast afferents (Figure 5; Bell et al., 1989; Bell, 1990). Subsequently, Bell and colleagues found that excitatory corollary discharge inputs to granule cells come from the medial juxtalobar nucleus (JLm) located at the anterior ventral margin of the ELL (Figure 5; Bell and von der Emde, 1995; Bell et al., 1995). The JLm receives inputs from the MCA, which also sends corollary discharge output to the nELL, indirectly through the sublemniscal nucleus (Figure 5; Bell and von der Emde, 1995). In summary, a corollary discharge that arises from the command nucleus facilitates sensory inputs in the mormyromast pathway. This increases the gain of mormyromast responses to self-generated EODs, which provides information about the surrounding environment.

### Echolocation in Bats

Do bats have a similar mechanism of corollary discharge that makes them more sensitive to the sounds they produce? In contrast to active electrolocation in mormyrids, in which they directly analyze self-generated electric pulses, bats do not use self-generated sounds directly, but rather compare information from the outgoing sound pulse and resulting echo to glean information about the surrounding environment.

Suga and Schlegel (1972) and Suga and Shimozawa (1974) demonstrated that the sensory response to a bat’s own call during vocalization was attenuated compared to the response to playback of the call during no vocalization. By contrast, Schuller provided evidence that a corollary discharge may enhance auditory responses to echoes of self-generated vocalizations in the greater horseshoe bat, *Rhinolophus ferrumequinum* (Schuller, 1979). He performed extracellular single-unit recordings from the inferior colliculus (IC) and compared responses to (1) playback of a simulated echo occurring just after a self-generated vocalization and (2) playback of both a simulated vocalization and simulated echo at rest. He found that IC neurons responded more strongly to (1) than to (2). Furthermore, the facilitation of echo responses by self-generated vocalization vanished when the phantom echo was delivered at delays longer than 60 ms. This indicates that the enhancement of echo responses has a specific time window and that the bat might have a detection range limit of ∼10 m distance (Neuweiler, 2003).

The neural source of vocalization-related enhancement in bat echolocation remains to be determined. Suga and Shimozawa suggested sensory attenuation by vocalization occurred in the nucleus of the lateral leminiscus, but it is not known whether enhancement of echo processing also happens in this nucleus. In addition, Schuller noted that, because vocalization was elicited by electrical stimulation of the central gray matter of the midbrain, this facilitation by self-vocalization might be different from natural echolocation during free flight (Schuller, 1979; Nachtigal and Schuller, 2014). Recently, telemetry neural recording techniques were developed and used for recording from the hippocampus and the superior colliculus during free flight in bats (Yartsev and Ulanovsky, 2013; Kothari et al., 2018). In the future, these techniques may be used to reveal the nature of motor-related enhancement under more natural conditions. Also, it remains to be determined whether corollary discharge, sensory feedback, or both are involved in vocalization-related enhancement of echo responses (see Figure 3).

## Corollary Discharge Is Used to Generate Predictions and Memories

Corollary discharges discussed so far are wired robustly to sensory circuits to suppress or facilitate sensory responses to self-generated stimuli. However, canceling predicted sensory inputs caused by own behavior does not always mean complete inhibition, especially when the motor act and the reafferent response patterns are complex and long lasting. In this case, complete inhibition would render the animal completely insensitive to sensory stimulation for a prolonged period of time. Instead, von Holst and Mittelstaedt suggested that an internal signal representing a “negative image” (i.e., efference copy) of the expected reafference would act to cancel the sensory consequences of own behavior while maintaining sensitivity to exafferent stimuli (von Holst and Mittelstaedt, 1950). Further, the internal prediction should be plastic so that it can be updated in response to environmental change because such change can alter the reafferent input in response to own behavior. Such a modifiable efference copy was first discovered in the passive electrosensory system of mormyrid fish.

### Negative Image Predicts Reafferent Input in the Passive Electrosensory System of Mormyrids

Mormyrid fish can detect and orient to the low-frequency electric signals generated by aquatic organisms such as insects and worms (Figure 1). This behavior is called passive electrolocation, and is shared with other animals that have electrosensation such as sharks and rays (Kalmijn, 1971). However, mormyrid fish face a difficult task for such electrolocation because they produce EODs that are much larger in amplitude than the weak, low-frequency signals generated by their prey. Indeed, the primary afferents of ampullary receptors that detect low-frequency electric fields respond to self-generated EODs with a long-lasting (∼100 ms), complex, multiphasic response (Bell and Russell, 1978). If a corollary discharge completely inhibited self-generated responses as in the Knollenorgan pathway, it would mask behaviorally important signals for a prolonged period of time.

**FIGURE 7:**
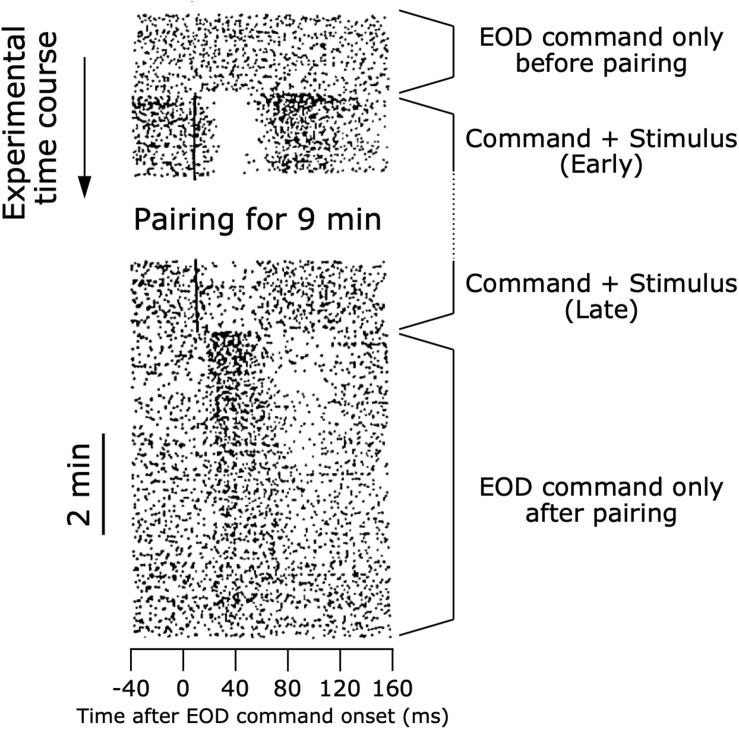
Modifiable efference copy in an ELL neuron. Raster shows responses of a cell in the ampullary region of the ELL. Each dot represents a spike, and each row shows the spiking activity aligned to each EOD command onset (see also Figure 4). At the beginning of the experiment, the EOD command alone did not affect the spiking activity of the cell. When an electrosensory stimulus was paired with the EOD command, the stimulus initially evoked a pause-burst spiking response of the cell. After several minutes of paring, the response to the electrosensory stimulus decreased dramatically. Upon removal of the electrosensory stimulus, the cell then showed a response to the EOD command alone. The shape of this response to the EOD command just after pairing represented a negative image of the initial response to electrosensory stimulation at the beginning of pairing. As time passed, the cell no longer responded to the EOD command alone. Modified from Bell (1989).

Bell examined how a corollary discharge solves this problem in the ampullary electrosensory pathway by obtaining unit recordings from the ampullary region of ELL (Bell, 1981). When an electric pulse stimulus triggered by the EOD command was delivered to a curarized fish (Figure 4A), ELL neurons initially responded with long-lasting, complex changes in spike rate similar to the responses of primary afferents (Figure 7). After repeated presentation of the stimulus, however, the sensory responses of ELL neurons decreased markedly (Figure 7). Next, Bell removed the paired electric pulse stimulus and observed the ELL neurons’ responses to the EOD command alone (Figure 7). The ELL neurons now showed a response to the EOD command, even though they showed no response to the command before presenting the paired stimulus (Figure 7). Remarkably, the shape of this response to the EOD command after removal of the stimulus was similar to an inverted version of the initial sensory response to the electric pulse stimulus (Figure 7). This result strongly indicated that a corollary discharge conveyed a negative image to subtract the predicted reafferent responses to the fish’s own EOD, and that this negative image was generated through plasticity. Indeed, the strong responses of ELL neurons to the command alone that was observed just after removing the paired stimulus gradually dissipated with time, reflecting a constant process of updating the negative image as the sensory consequences of behavior changed.

**FIGURE 8:**
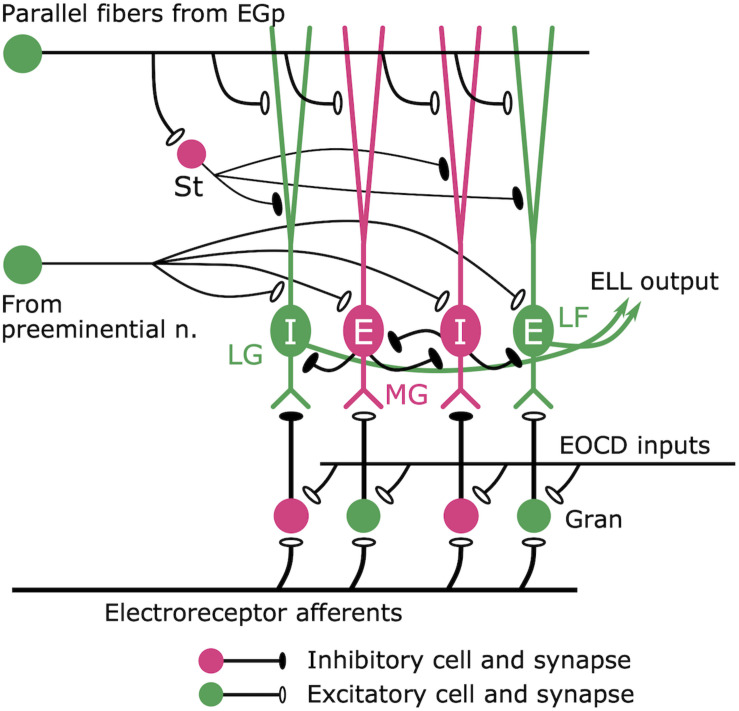
Cerebellum-like circuit in the ELL cortex. Mormyromast and ampullary afferents terminate on granule cells (gran). In the mormyromast region of ELL, the granule cells receive precisely timed electric organ corollary discharge (EOCD) input. However, the ampullary region lacks this input (not shown here). The granule cells provide both excitatory and inhibitory outputs to the downstream neurons. The large fusiform (LF) cells and the lateral ganglion (LG) cells receive excitatory and inhibitory inputs from granule cells, respectively. Medium ganglion (MG) cells are Purkinje-like cells that receive sensory inputs from granule cells and provide major inhibitory inputs to the LF cells and LG cells, which send their outputs to higher centers. E cells are excited by an increase in afferent activity, while I cells are inhibited. Parallel fibers provide corollary discharge input to the apical dendrites of MG cells, LF cells, and LG cells directly and indirectly via inhibitory stellate (St) cells. In addition, the preeminential nucleus provides electrosensory feedback to MG cells, LF cells, and LG cells. Modified from Sawtell et al. (2005).

What is the neural circuit mediating this efference copy? Maler first pointed out an interesting anatomical feature of the ELL: similarities to the cerebellum of mammals (Maler, 1973; Figure 8). The ELL has Purkinje-like GABAergic neurons (called MG cells) that receive inputs from primary electrosensory afferents via granule cells in a deep layer, as well as inputs from parallel fibers in a superficial molecular layer (Figure 8). Libouban and Szabo (1977) described a pathway from the paratrigeminal command-associated nucleus (PCA) to the eminentia granularis posterior (EGp), whose axons form the parallel fibers in the ELL (Figure 5). In addition, Bell, Libouban, and Szabo found that the PCA receives inputs from BCA (Bell et al., 1983; Figure 5). These anatomical studies suggested that the parallel fibers convey corollary discharge information and that the MG cells integrate inputs from primary electrosensory afferents and this corollary discharge pathway.

Bell et al. (1993, 1997) investigated how negative images emerge in this cerebellum-like circuit. Using intracellular recording, they found that MG cells produce two types of spikes: broad spikes that occur in the apical dendrites that receive corollary-discharge input and narrow spikes that occur in axons (Bell et al., 1993). They found that paring a broad spike evoked by current injection with the EOD command could induce synaptic plasticity at the parallel fiber synapses (Bell et al., 1993). Further *in vitro* study revealed an anti-Hebbian rule to this synaptic plasticity: excitatory postsynaptic potentials (EPSPs) evoked by electric stimulation of parallel fibers preceding broad spikes induced synaptic depression, whereas EPSPs following broad spikes induced synaptic potentiation (Bell et al., 1997). These results demonstrated that negative images were generated at the synapses between parallel fibers and Purkinje-like cells through spike-timing-dependent plasticity with an anti-Hebbian learning rule. This was among the first demonstrations of spike-timing-dependent plasticity in any neural circuit (Markram et al., 2011).

In order to form a negative image that lasts long enough to cancel reafferent inputs from ampullary afferents, parallel fibers need to provide temporally variable inputs that cover the duration of afferent responses. This property of parallel fibers had been assumed for a long time, but it had not been directly tested, and the underlying mechanisms for temporal dispersion remained unknown. Sawtell, Kennedy et al. (2014) found that EGp received a brief corollary discharge input and that the parallel fibers indeed provide such a temporal basis, which was mediated by relaying interneurons in EGp called unipolar brush cells. This finding linked the corollary discharge circuit to synaptic plasticity to describe the formation of long-lasting negative images.

Note that a similar cancelation of predictive signals was also found in the mormyromast pathway (Bell and Grant, 1992). While afferents from ampullary receptors and mormyromast receptors innervate different regions of the ELL cortex, MG cells of both regions receive corollary discharge inputs from parallel fibers and afferent inputs from granule cells (Figure 5). It is thought that the same process mediating negative image formation in the ampullary region of ELL cortex is also occurring in the cerebellum-like circuit of the mormyromast region of ELL cortex. This serves to cancel predicted reafferent responses in the active electrolocation pathway, so that the system only responds to novel, unexpected sensory inputs.

### Cerebellum-Like Circuits Mediate Subtraction of Self-Generated Inputs

After the discovery of modifiable internal predictions was made in the mormyrid ELL, many similar mechanisms were found in other cerebellum-like structures across various sensory modalities and species (reviewed in Bell et al., 2008). For example, the skate *Raja erinacea* is a cartilaginous fish that has a low-frequency, passive electrosensory system. Similar to mormyrid fish, own movements such as respiration strongly affect electrosensory processing. This reafference problem is solved by a cerebellum-like circuit in the dorsal octovolateral nucleus (DON) where primary electrosensory afferents terminate (Bodznick and Northcutt, 1980; Montgomery, 1984; New and Bodznick, 1990). Furthermore, similar to the passive electrosensory system in mormyrids, this cancelation appears to be modifiable through learning as shown by an experiment using paired stimulation (Montgomery and Bodznick, 1994). A similar cancelation phenomenon was found in the medial octovolateral nucleus (MON) of scorpion fish, *Scorpoena papillosus*, which is the first sensory center with a cerebellum-like structure in the mechanosensory lateral line system (Montgomery and Bodznick, 1994).

Another example of a cerebellum-like circuit is the dorsal cochlear nucleus (DCN) in the auditory pathway of mammals. Own behaviors including vocalization, chewing, licking, and other movements of body parts have predictable auditory consequences that may disrupt auditory processing. The DCN directly receives primary auditory afferents from the cochlea in the deep layer and also receives motor-related inputs including corollary discharge information via parallel fibers in the molecular layer (Oertel and Young, 2004). Like MG cells in the mormyrid ELL, *in vitro* studies showed plasticity at the synapse between the parallel fibers and the GABAergic Purkinje-like cells (called cartwheel cells) that follows an anti-Hebbian rule (Tzounopoulos et al., 2004, 2007). Until recently, whether the cerebellum-like circuit in the DCN works to subtract predictable signals was untested. Singla et al. (2017) developed a unique experiment with mice to directly test this hypothesis, in which they delivered auditory stimulation paired with licking behavior. They found that DCN neurons reliably encoded external auditory stimuli even during licking. Moreover, DCN neurons reduced responsiveness to auditory stimuli that were repeatedly temporally correlated with licking, suggesting that the DCN circuit creates adaptive filters for canceling self-generated sound through learning, much like the generation of negative images in the ELL of mormyrids.

Modifications that adapt to the sensory consequences of own behavior in the cerebellum-like circuits discussed here are not necessarily due to corollary discharges, and could be due to sensory feedback (see Figure 3). However, these studies highlight how the cerebellum-like circuit in mormyrid ELL provided general insight into how various circuits solve the problem of canceling the predictable sensory consequences of own behavior.

### Subtraction of Expected Signals in Primate Vestibular Processing

Using vestibular organs of the inner ear, the vestibular system can detect head motion, including rotational and translational velocities relative to space. This sensory information is used for maintaining posture, perceiving self-motion, and computing spatial orientation. As with other sensory modalities, distinguishing self-generated from external stimuli is important for these functions.

All afferent fibers from the vestibular organs project to the vestibular nucleus and terminate on two categories of neurons: vestibulo-ocular reflex (VOR) neurons and vestibular-only (VO) neurons (reviewed in Cullen, 2012). While the vestibular afferents encode vestibular stimuli caused by both external and self-generated changes in a similar way, VO neurons do not provide reliable information about active head movements (Boyle et al., 1996; McCrea et al., 1999; Roy and Cullen, 2001; Cullen and Minor, 2002). This suggested that a corollary discharge from the neck motor command directly inhibits the VO neurons, but this was not supported experimentally (Roy and Cullen, 2004). Alternatively, Roy and Cullen proposed a more interesting mechanism: an inhibitory neck proprioceptive signal is gated in only when the actual activation of neck proprioceptors matches an internal prediction (corollary discharge) of the consequence of head motion (Roy and Cullen, 2004).

Next, Cullen et al. (2011) were interested in where and how internal predictions and actual neck proprioceptive signals meet. They focused on the rostral fastigial nucleus (rFN) in the deep cerebellum. The rFN receives descending projections from the anterior vermis, a region of the cerebellum that receives direct projections from cortical structures involved in producing head and neck movement (Batton et al., 1977; Yamada and Noda, 1987; Alstermark et al., 1992a, b; Cullen et al., 2011). That is, the rFN would receive a corollary discharge of neck motor commands. In addition, the rFN integrates vestibular and proprioceptive inputs and contains unimodal neurons (vestibular only) and bimodal neurons (vestibular and proprioceptive) (Brooks and Cullen, 2009). Furthermore, Brooks and Cullen showed, during active movement, that unimodal neurons encode unexpected head motions whereas bimodal neurons encode unexpected body motion (Brooks and Cullen, 2013). This result indicated that information of expected motion was subtracted in the rFN. Moreover, Brooks et al. (2015) found that loading the monkey’s movement, which resulted in a difference between estimated sensory consequences of own behavior and actual sensory consequences, altered this internal prediction. Trial-by-trial changes in the neuronal response were gradual and consistent with the resultant behavioral learning. This describes a similar process to generating negative images in mormyrid fish.

### Predictive Visual Representation During Saccades in Primates

A milestone in the study of corollary discharge in predictive sensory coding would be a series of studies on visual representation during saccades in primates. Duhamel et al. (1992) addressed how eye movement affects the receptive fields of neurons, i.e., the region of space that can elicit a visual response. They recorded neural activities from the lateral intraparietal area (LIP) of rhesus macaques, *Maccaca mulatta*. They found that LIP neurons respond to a visual stimulus in their receptive field with a 70 ms latency. Next, a visual stimulus was positioned so that it would be in the receptive field after the monkey completed a saccade. Although the neurons would be expected to start firing 70 ms after the eye movement brought the stimulus into the receptive field, Duhamel et al. (1992) found that the cells started responding 80 ms before the saccade was initiated. That is, the receptive field location shifted before the eye movement. A similar receptive field shift was also found in the frontal eye field (FEF) of the frontal cortex (Umeno and Goldberg, 1997). These results suggest that a corollary discharge conveying internal predictions accurately adjusts the receptive field of LIP and FEF visual neurons in anticipation of intended eye movements.

Sommer and Wurtz investigated the neural pathway that mediates visual stability by corollary discharge. The candidate source of corollary discharge was the superior colliculus (SC) because the SC contains neurons that fire just before initiating a saccade, suggesting it is a motor control center for eye movement (Sparks and Hartwich-Young, 1989). Anatomical research showed a neural pathway from the SC to the FEF via the mediodorsal nucleus (MD) of the thalamus, suggesting this pathway could convey corollary discharges related to eye movement (Lynch et al., 1994). Sommer and Wurtz (2002, 2004) found that this pathway encoded the vector of upcoming eye movements and that inactivation of this pathway impaired a corollary discharge-related behavioral task (double-step saccade task). Furthermore, they found that the shift of receptive field in the FEF neurons before upcoming eye movements was impaired by interrupting the corollary discharge signal from the MD (Sommer and Wurtz, 2006). This result demonstrated the causality of corollary discharge input from the MD in signaling the vector of intended eye movement to shift the receptive fields of FEF neurons.

### Internal Prediction Mediates Sensorimotor Learning in Songbirds

Songbirds acquire specific song patterns through vocal learning during development. Vocal learning consists of three phases including (1) sensory learning: modifying the internal template of own song based on the songs of one or more tutors; (2) sensorimotor learning: matching own song performance to the internal template; and (3) crystallization: establishment of fixed, mature song patterns (Marler, 1964; Konishi, 1965). The neural mechanisms underlying vocal learning in songbirds have attracted many neuroscientists because of striking similarities to the development of human speech (Doupe and Kuhl, 1999).

An important question in vocal learning was how the nervous system can compare auditory feedback from own song with the internal template during sensorimotor learning. Using modeling studies, Troyer and Doupe proposed that a corollary discharge plays an essential role in comparing the tutor’s song stored in its memory to actual auditory feedback (Troyer and Doupe, 2000a, b). According to this hypothesis, when the bird vocalizes, a corollary discharge representing an internal prediction of the template was emitted and compared with the actual feedback. The errors between the template and the sensory consequences of vocalization were thought to be corrected by a repeating cycle including vocal production and adjustment of the motor program. To date, however, the nature of this corollary discharge remains controversial.

The neural pathways mediating bird song production and learning have been well characterized (Nottebohm, 2005). The telencephalic nucleus called the high vocal center (HVC) plays an important role in both song production and learning and is a source of two important pathways: (1) posterior descending pathway (PDP) necessary for both learning and production, and (2) anterior forebrain pathway (AFP) necessary for learning only (Nottebohm, 2005). Prather et al. (2008) found that the first projection neuron in the AFP (i.e., HVC–>Area X) responds both to own song production and auditory feedback with the same latency. This feature is similar to mirror neurons (Gallese et al., 1996) and also suggested this might be a suitable site for comparing feedback of own vocalization with the internal template. Furthermore, in recent years, a candidate corollary discharge pathway was identified (Roberts et al., 2017). Roberts et al. (2017) focused on another pathway from HVC to a small cluster of neurons (Avalanche, Av) embedded in the caudal mesopallium (CM), analog of the mammalian secondary auditory cortex (Akutagawa and Konishi, 2010). They identified a new type of projection neuron (HVC–>Av) that receives inputs from premotor neurons and transmits motor-related activity during song production. In addition, genetically ablating this type of neuron in juveniles disrupted vocal learning. Future studies should examine how the downstream circuit integrates internal predictions represented by a corollary discharge and actual sensory inputs and how the error signals are used to adjust motor programs.

## Corollary Discharge in the Patterning of Behavior

Although we have mostly discussed effects of corollary discharges on sensory processing thus far, corollary discharges have also been found to influence motor systems. For example, as discussed previously, corollary discharge inhibition regulates the temporal pattern generation of feeding behavior in sea slugs (Davis et al., 1973; Siegler et al., 1974; Gillette and Davis, 1977). Similar to this case, a corollary discharge pathway in mormyrid fish is also involved in generating rhythmic temporal patterns of EOD production.

### Temporal Pattern Generation of EOD Production in Mormyrids

Similar to other rhythmic behaviors such as locomotion and feeding, EOD production by mormyrid fish consists of variably rhythmic temporal patterns, which play an important role in communicating behavioral state (Carlson, 2002a). What neural circuitry governs EOD production? Bell et al. (1983) first identified the medullary command nucleus (CN) that controls EOD production, as well as corollary discharge pathways, using neuronal tracing with horseradish peroxidase. The CN projects to the medullary relay nucleus (MRN), which sends its output to the spinal electromotor neurons that innervate the electrocytes in the electric organ (EO), which produce the EOD (Bennett et al., 1967; Bell et al., 1983). The reasons why this nucleus was identified as a “command” nucleus are (1) its output is time-locked in a one-to-one manner with EOD generation, (2) it integrates major inputs from the mesencephalic precommand nucleus (PCN), minor inputs from the mesencephalic ventroposterior nucleus (VP), and unspecified inputs to the adjacent medial reticular formation, and (3) its neurons are interconnected by complex electronic coupling, resulting in the first occurrence of neuronal synchronization in the pathway (Bell et al., 1983; Elekes and Szabo, 1985; Grant et al., 1986).

How does the electromotor circuit generate variable temporal patterns of EOD production? The CN itself is not a pacemaker, rather it integrates descending inputs to decide whether or not to generate an EOD. von der Emde et al. (2000) first recorded neural activities from the PCN, and discovered two types of neurons. Neurons of one type fired in the moments leading up to fictive EOD production, but were inhibited immediately after each fictive EOD. Neurons of the second type fired bursts of spikes immediately after each fictive EOD, during the silent period of the first neuron type. This suggested that the first type of neuron was providing descending excitatory input to the CN, whereas the second type of neuron was relaying corollary discharge inhibition to the first type of neuron.

**FIGURE 9:**
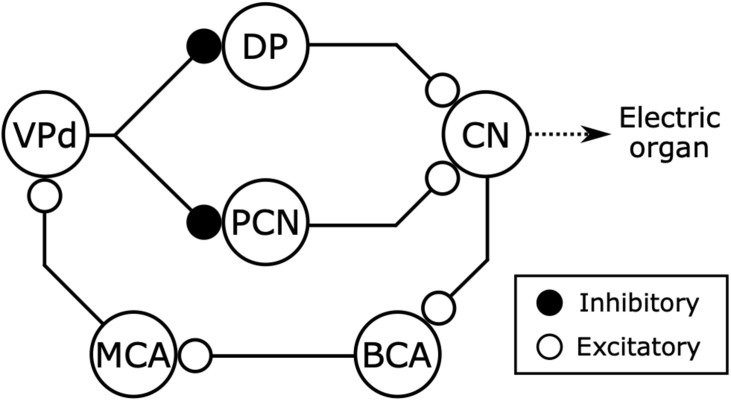
Electromotor network of mormyrids receives inhibitory feedback from the electric organ corollary discharge pathway. The command nucleus (CN) controls the timing of EOD production and also gives rise to a corollary discharge pathway including the bulbar command-associated nucleus (BCA) and mesencephalic command-associated nucleus (MCA) (see also Figure 5). The CN receives excitatory inputs from the thalamic dorsal posterior nucleus (DP) and the mesencephalic precommand nucleus (PCN). The DP and PCN both receive inhibitory input from the dorsal ventroposterior nucleus (VPd) of the torus semicircularis, which receives corollary discharge excitation from the MCA. Thus, the main sources of excitatory input to the CN receive inhibitory feedback from the corollary discharge pathway immediately following each EOD. Modified from Carlson (2003).

Carlson further studied the neuroanatomy of the electromotor system in the species *Brienomyrus brachyistius* (Carlson, 2002b). Carlson confirmed that the anatomical pathway was similar to that of *G. petersii* (Bell et al., 1983), and added important new findings: (1) In addition to PCN, the dorsal posterior nucleus of the thalamus (DP) also provides a major input to the CN; (2) VP has two distinct subdivisions, one dorsal (VPd) and one ventral (VPv); (3) VPv projects to the CN, DP, and PCN, whereas VPd projects only to DP and PCN; (4) VPd receives input from the corollary discharge pathway via MCA (Figure 9). These findings suggested that VPd neurons were the source of corollary discharge inhibition of PCN neurons first identified by von der Emde et al. (2000). Indeed, using single-unit extracellular recordings and pharmacological stimulation, Carlson and Hopkins demonstrated that VPd provides corollary discharge inhibition to DP and PCN and that disinhibition increases the EOD rate (Carlson, 2003; Carlson and Hopkins, 2004). Thus, recurrent inhibition of premotor circuits by a corollary discharge can act to regulate rhythmic motor output.

Both DP and VPd are connected reciprocally with the optic tectum (Wullimann and Northcutt, 1990; Carlson, 2002b), which is considered a primary sensorimotor hub (Meek and Nieuwenhuys, 1998). This suggests that the EOD command network integrates sensory information and inhibitory feedback from a corollary discharge to generate rhythmic EOD patterns, much like the feeding circuit found in the sea slug *Pleurobranchea* (Davis et al., 1973; Gillette and Davis, 1977). Thus, a similar integration of corollary discharge feedback and sensory input may shape rhythmic motor output across invertebrate and vertebrate species.

### Corollary Discharge Mediates Motor Coupling in Larval Tadpoles

Another important finding on the role of corollary discharge in governing behavioral pattern generation comes from a series of studies on the link between spinal locomotion circuits and eye-movement circuits in larval tadpoles. Locomotion such as swimming results in both body movements and head movements, which may greatly disrupt visual perception. To stabilize their visual world, aquatic animals move their eyes in conjunction with tail movements to minimize retinal image slip (Lyon, 1900; Harris, 1965; Easter and Johns, 1974; Chagnaud et al., 2012). This motor coupling could be explained by the concerted actions of visuo-vestibular and proprioceptive reflexes (Angelaki and Hess, 2005). However, earlier behavioral studies suggested that such sensorimotor transformations would be relatively slow due to the filtering characteristics of the sensory periphery (Lyon, 1900; Harris, 1965; Easter and Johns, 1974; Chagnaud et al., 2012). Instead, central, motor-related signals may inform eye-movement circuits about ongoing locomotor patterns.

Stehouwer first demonstrated this possibility using a preparation of an isolated central nervous system including only the nerves innervating extraocular muscles of the eyes of a larval bullfrog (*Rana catesbeiana*) (Stehouwer, 1987). The reduced *in vitro* preparation enabled recording from motor neurons that innervate extraocular muscles during fictive swimming, which was indicated by burst activity of axial motor neurons in the spinal cord. By isolating the central nervous system and eliminating movement, the effects of sensory feedback, such as vestibular and proprioceptive inputs, were eliminated (see Figure 3C). He found that burst activities from motor neurons mediating eye movement were phase-locked to burst activities associated with fictive swimming. This result suggested that motor coupling between swimming and eye movement depends on intrinsic communication between the brain and spinal cord.

Lambert et al. (2012) later examined the link between spinal swimming circuitry and eye-movement circuitry using larval *Xenopus laevis*. They removed other supraspinal areas such as the midbrain reticular formation, cerebellum, or vestibular nucleus from the *in vitro* preparation, and found that this motor coupling remained intact. In addition, they delineated an ascending pathway from the spinal cord to eye-movement circuitry based on anatomical evidence. These results demonstrated that a corollary discharge from the spinal swimming circuit directly regulates the eye-movement used for gaze stabilization.

These studies established the novel concept that corollary discharges can affect other motor circuits in addition to sensory processing (reviewed in Straka et al., 2018). Such corollary discharge function is not likely limited to swimming-extraocular motor coupling in tadpoles. For example in cats, there are pathways that convey motor information during scratching from the spinal cord to the cerebellum (Arshavsky et al., 1978a, b; Martínez-Silva et al., 2014). It is speculated that the corollary discharge feedback may be used to compare and adjust the precision of movements according to environmental demands (Morton and Bastian, 2004), although there is no behavioral evidence as of yet. In addition, in mormyrid fish, corollary discharge related potentials are also found in the cerebellum (Bennett and Steinbach, 1969), but their function remains unknown.

## Evolution of Corollary Discharge Function

As we have described, corollary discharge is found across various sensory modalities and species. While mechanisms underlying corollary discharge have been extensively studied in select species, little is known about the evolution of corollary discharge circuits and mechanisms. How have animals acquired novel corollary discharge functions through evolution? How have corollary discharges evolved along with evolutionary change in behavior? Comparative studies of weakly electric mormyrid fish may provide answers to these questions.

Does acquiring electrogenesis mean emergence of corollary discharge function? The answer seems to be no. The ability of electrogenesis has evolved at least 6 times independently in fish (Gallant et al., 2014). The electric fishes can be categorized into two groups: wave-type fish that generate continuous, quasi-sinusoidal EODs in which the interval between each EOD is approximately equal to the duration of each EOD; and pulse-type fish that generate discrete EODs with longer periods of silence between them. While all mormyrids generate pulse-type EODs, the closest relative to mormyrids, *Gymnarchus niloticus*, generates a wave-type EOD. Recently, we demonstrated that an electric organ corollary discharge seems to exist in all species of mormyrids (Vélez and Carlson, 2016), but *Gymnarchus* appears to lack an electric organ corollary discharge pathway (Kawasaki, 1993, 1994). This suggests that an electric organ corollary discharge pathway evolved with the origin of pulse-type EODs in mormyrids. However, in the distantly related gymnotiform electric fish, it appears that neither wave-type nor pulse-type species have an electric organ corollary discharge pathway (Kawasaki and Heiligenberg, 1990; Keller et al., 1990; Heiligenberg, 1991; Heiligenberg and Kawasaki, 1992; Kennedy and Heiligenberg, 1994). Mormyrids generate EODs at much more variable rates than pulse-type gymnotiforms (Kawasaki and Heiligenberg, 1990; Carlson, 2002a). It may be that a corollary discharge is important for signaling the timing of EOD production in fish that generate EODs with greater irregularity, that is with less predictability. Regardless, these findings reveal that evolving electrogenesis does not always mean acquiring a novel electric organ corollary discharge pathway.

Nearly all detailed studies of corollary discharge circuitry and mechanisms in mormyrids have focused on one species, *Gnathonemus petersii.* However, EODs have diversified extensively across the mormyrid family, especially in duration, which varies across species from 0.1 to over 10 ms (Hopkins, 1999). How does corollary discharge function vary with these electric signals? Recently, our group compared corollary discharge inhibition in the communication pathway among several species with varying EOD durations (Fukutomi and Carlson, 2020). We found that fish with long-duration EODs have delayed corollary discharge inhibition of the nELL and that this time-shifted corollary discharge optimally blocks electrosensory responses to the fish’s own EOD (Fukutomi and Carlson, 2020). This suggests that corollary discharge mechanisms coevolve along with the evolution of communication signals, but the underlying mechanisms for shifting this inhibitory delay remain unknown.

## Concluding Remark

**FIGURE 10:**
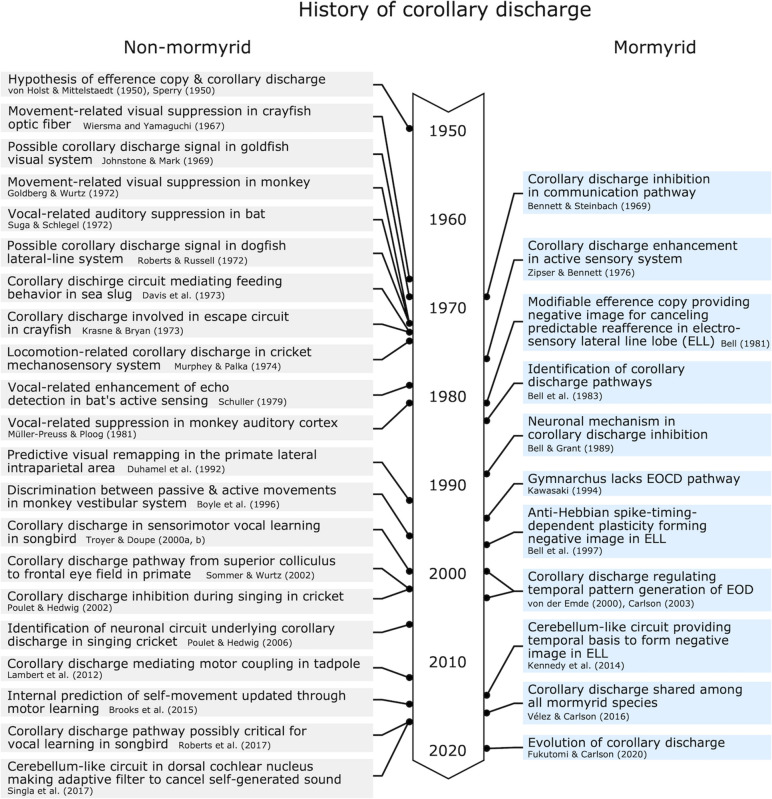
Chronological table for major discoveries related to corollary discharge mechanisms.

Here, we discussed how corollary discharge mechanisms have been understood in a historical context, with a focus on the study of mormyrid weakly electric fish (Figure 10). Because dysfunction of corollary discharge may be related to psychiatric diseases such as schizophrenia in humans (Ford et al., 2001), studying corollary discharge mechanisms is important in medical science as well as basic science. Since the concepts of corollary discharge and efference copy were proposed in 1950, studies in mormyrids have pioneered our understanding of the underlying circuitry and mechanisms. Although many animals including humans have neither electrosensory systems nor the ability to actively generate electric fields, these findings in mormyrids have provided insights that have led to general principles of corollary discharge function and mechanism, including inhibition in communication, enhancement in active sensing, modifiable efference copies involved in learning and sensorimotor integration, and feedback to premotor centers for regulating behavioral output. The generality of these principals has been supported by numerous studies on a diversity of species and sensory systems.

## Author Contributions

Both authors conceptualized the review and wrote the manuscript.

## Conflict of Interest

The authors declare that the research was conducted in the absence of any commercial or financial relationships that could be construed as a potential conflict of interest.

The handling editor declared a past collaboration with one of the authors BC.

## References

[B1] Akutagawa E. Konishi M. (2010). New brain pathways found in the vocal control system of a songbird. *J. Comp. Neurol.* 518 3086–3100. 10.1002/cne.22383 20533361

[B2] Alexander G. E. Newman J. D. Symmes D. (1976). Convergence of prefrontal and acoustic inputs upon neurons in the superior temporal gyrus of the awake squirrel monkey. *Brain Res.* 116 334–338. 10.1016/0006-8993(76)90913-6 824022

[B3] Alstermark B. Pinter M. J. Sasaki S. (1992a). Descending pathways mediating disynaptic excitation of dorsal neck motoneurones in the cat: facilitatory interactions. *Neurosci. Res.* 15 32–41. 10.1016/0168-0102(92)90015-5 1336583

[B4] Alstermark B. Pinter M. J. Sasaki S. (1992b). Descending pathways mediating disynaptic excitation of dorsal neck motoneurones in the cat: brain stem relay. *Neurosci. Res.* 15 42–57. 10.1016/0168-0102(92)90016-6 1336584

[B5] Angelaki D. E. Hess B. J. M. (2005). Self-motion-induced eye movements: effects on visual acuity and navigation. *Nat. Rev. Neurosci.* 6 966–976. 10.1038/nrn1804 16340956

[B6] Arshavsky I. Gelfand I. M. Orlovsky G. N. Pavlova G. A. (1978a). Messages conveyed by spinocerebellar pathways during scratching in the cat. I. Activity of neurons of the lateral reticular nucleus. *Brain Res.* 151 479–491. 10.1016/0006-8993(78)91081-8 667626

[B7] Arshavsky I. Gelfand I. M. Orlovsky G. N. Pavlova G. A. (1978b). Messages conveyed by spinocerebellar pathways during scratching in the cat. II. Activity of neurons of the ventral spinocerebellar tract. *Brain Res.* 151 493–506. 10.1016/0006-8993(78)91082-x 667627

[B8] Baker C. A. Kohashi T. Lyons-Warren A. M. Ma X. Carlson B. A. (2013). Multiplexed temporal coding of electric communication signals in mormyrid fishes. *J. Exp. Biol.* 216 2365–2379. 10.1242/jeb.082289 23761462 PMC3680504

[B9] Baker C. A. Ma L. Casareale C. R. Carlson B. A. (2016). Behavioral and single-neuron sensitivity to millisecond variations in temporally patterned communication signals. *J. Neurosci.* 36 8985–9000. 10.1523/JNEUROSCI.0648-16.2016 27559179 PMC4995309

[B10] Batton R. R. Jayaraman A. Ruggiero D. Carpenter M. B. (1977). Fastigial efferent projections in the monkey: an autoradiographic study. *J. Comp. Neurol.* 174 281–305. 10.1002/cne.901740206 68041

[B11] Bell C. Dunn K. Hall C. Caputi A. (1995). Electric organ corollary discharge pathways in mormyrid fish. *J. Comp. Physiol. A* 177 449–462. 10.1007/bf00187481

[B12] Bell C. von der Emde G. (1995). Electric organ corollary discharge pathways in mormyrid fish. *J. Comp. Physiol. A* 177 463–479. 10.1007/bf00187482

[B13] Bell C. C. (1981). An efference copy which is modified by reafferent input. *Science* 214 450–453. 10.1126/science.7291985 7291985

[B14] Bell C. C. (1989). Sensory coding and corollary discharge effects in mormyrid electric fish. *J. Exp. Biol.* 146 229–253. 2689564 10.1242/jeb.146.1.229

[B15] Bell C. C. (1990). Mormyromast electroreceptor organs and their afferent fibers in mormyrid fish. II. Intra-axonal recordings show initial stages of central processing. *J. Neurophysiol.* 63 303–318. 10.1152/jn.1990.63.2.303 2313347

[B16] Bell C. C. Caputi A. Grant K. Serrier J. (1993). Storage of a sensory pattern by anti-Hebbian synaptic plasticity in an electric fish. *Proc. Natl. Acad. Sci. U.S.A.* 90 4650–4654. 10.1073/pnas.90.10.4650 8506312 PMC46570

[B17] Bell C. C. Finger T. E. Russell C. J. (1981). Central connections of the posterior lateral line lobe in mormyrid fish. *Exp. Brain Res.* 42 9–22. 10.1007/BF00235724 6163655

[B18] Bell C. C. Grant K. (1989). Corollary discharge inhibition and preservation of temporal information in a sensory nucleus of mormyrid electric fish. *J. Neurosci.* 9 1029–1044. 10.1523/jneurosci.09-03-01029.1989 2926477 PMC6569966

[B19] Bell C. C. Grant K. (1992). Sensory processing and corollary discharge effects in mormyromast regions of mormyrid electrosensory lobe. II. Cell types and corollary discharge plasticity. *J. Neurophysiol.* 68 859–875. 10.1152/jn.1992.68.3.859 1432053

[B20] Bell C. C. Han V. Sawtell N. B. (2008). Cerebellum-like structures and their implications for cerebellar function. *Annu. Rev. Neurosci.* 31 1–24. 10.1146/annurev.neuro.30.051606.094225 18275284

[B21] Bell C. C. Han V. Z. Sugawara Y. Grant K. (1997). Synaptic plasticity in a cerebellum-like structure depends on temporal order. *Nature* 387 278–281. 10.1038/387278a0 9153391

[B22] Bell C. C. Libouban S. Szabo T. (1983). Pathways of the electric organ discharge command and its corollary discharges in mormyrid fish. *J. Comp. Neurol.* 216 327–338. 10.1002/cne.902160309 6306068

[B23] Bell C. C. Russell C. J. (1978). Effect of electric organ discharge on ampullary receptors in a mormyrid. *Brain Res.* 145 85–96. 10.1016/0006-8993(78)90798-9 638785

[B24] Bell C. C. Zakon H. Finger T. E. (1989). Mormyromast electroreceptor organs and their afferent fibers in mormyrid fish: I. Morphology. *J. Comp. Neurol.* 286 391–407. 10.1002/cne.902860309 2768566

[B25] Bennett M. V. Pappas G. D. Aljure E. Nakajima Y. (1967). Physiology and ultrastructure of electrotonic junctions. II. Spinal and medullary electromotor nuclei in mormyrid fish. *J. Neurophysiol.* 30 180–208. 10.1152/jn.1967.30.2.180 4167209

[B26] Bennett M. V. L. (1965). Electroreceptors in mormyrids. *Cold. Spring Harb. Sym.* 30 245–262. 10.1101/sqb.1965.030.01.027 5219479

[B27] Bennett M. V. L. Steinbach A. B. (1969). “Influence of electric organ control system on electrosensory afferent pathways in Mormyrids,” in *Neurobiology of Cerebellar Evolution and Development*, ed. Llinás R. (Chicago, IL: American Medical Association), 207–214.

[B28] Bodznick D. Northcutt R. G. (1980). Segregation of electro- and mechanoreceptive inputs to the elasmobranch medulla. *Brain Res.* 195 313–321. 10.1016/0006-8993(80)90067-0 7397504

[B29] Boyle R. Belton T. McCrea R. A. (1996). Responses of identified vestibulospinal neurons to voluntary eye and head movements in the squirrel monkey. *Ann. N.Y. Acad. Sci.* 781 244–263. 10.1111/j.1749-6632.1996.tb15704.x 8694418

[B30] Brooks J. X. Carriot J. Cullen K. E. (2015). Learning to expect the unexpected: rapid updating in primate cerebellum during voluntary self-motion. *Nat. Neurosci.* 18 1310–1317. 10.1038/nn.4077 26237366 PMC6102711

[B31] Brooks J. X. Cullen K. E. (2009). Multimodal integration in rostral fastigial nucleus provides an estimate of body movement. *J. Neurosci.* 29 10499–10511. 10.1523/jneurosci.1937-09.2009 19710303 PMC3311469

[B32] Brooks J. X. Cullen K. E. (2013). The primate cerebellum selectively encodes unexpected self-motion. *Curr. Biol.* 23 947–955. 10.1016/j.cub.2013.04.029 23684973 PMC6100740

[B33] Carlson B. A. (2002a). Electric signaling behavior and the mechanisms of electric organ discharge production in mormyrid fish. *J. Physiol. Paris* 96 405–419. 10.1016/S0928-4257(03)00019-6 14692489

[B34] Carlson B. A. (2002b). Neuroanatomy of the mormyrid electromotor control system. *J. Comp. Neurol.* 454 440–455. 10.1002/cne.10462 12455008

[B35] Carlson B. A. (2003). Single-unit activity patterns in nuclei that control the electromotor command nucleus during spontaneous electric signal production in the mormyrid *Brienomyrus brachyistius*. *J. Neurosci.* 23 10128–10136. 10.1523/jneurosci.23-31-10128.2003 14602829 PMC6740866

[B36] Carlson B. A. (2009a). “Reafferent control in electric communication,” in *Encyclopedia of Neuroscience*, eds Binder M. D. Hirokawa N. Windhorst U. Hirsch M. C. (Berlin: Springer). 10.1007/978-3-540-29678-2_4945

[B37] Carlson B. A. (2009b). Temporal-pattern recognition by single neurons in a sensory pathway devoted to social communication behavior. *J. Neurosci.* 29 9417–9428. 10.1523/JNEUROSCI.1980-09.2009 19641105 PMC2819125

[B38] Carlson B. A. Hopkins C. D. (2004). Central control of electric signaling behavior in the mormyrid *Brienomyrus brachyistius*: segregation of behavior-specific inputs and the role of modifiable recurrent inhibition. *J. Exp. Biol.* 207 1073–1084. 10.1242/jeb.00851 14978050

[B39] Casas J. Dangles O. (2010). Physical ecology of fluid flow sensing in arthropods. *Annu. Rev. Entomol.* 55 505–520. 10.1146/annurev-ento-112408-085342 19743914

[B40] Chagnaud B. P. Simmers J. Straka H. (2012). Predictability of visual perturbation during locomotion: implications for corrective efference copy signaling. *Biol. Cybern.* 106 669–679. 10.1007/s00422-012-0528-0 23179256

[B41] Crapse T. B. Sommer M. A. (2008). Corollary discharge across the animal kingdom. *Nat. Rev. Neurosci.* 9 587–600. 10.1038/nrn2457 18641666 PMC5153363

[B42] Creutzfeldt O. Ojemann G. Lettich E. (1989). Neuronal activity in the human lateral temporal lobe. *Exp. Brain Res.* 77 476–489. 10.1007/bf00249601 2806442

[B43] Cullen K. E. (2004). Sensory signals during active versus passive movement. *Curr. Opin. Neurobiol.* 14 698–706. 10.1016/j.conb.2004.10.002 15582371

[B44] Cullen K. E. (2012). The vestibular system: multimodal integration and encoding of self-motion for motor control. *Trends Neurosci.* 35 185–196. 10.1016/j.tins.2011.12.001 22245372 PMC4000483

[B45] Cullen K. E. Brooks J. X. Jamali M. Carriot J. Massot C. (2011). Internal models of self-motion: computations that suppress vestibular reafference in early vestibular processing. *Exp. Brain Res.* 210 377–388. 10.1007/s00221-011-2555-9 21286693

[B46] Cullen K. E. Minor L. B. (2002). Semicircular canal afferents similarly encode active and passive head-on-body rotations: implications for the role of vestibular efference. *J. Neurosci.* 22:RC226. 10.1523/jneurosci.22-11-j0002.2002 12040085 PMC6758814

[B47] Davis W. J. Mpitsos G. J. Pinneo J. M. Ram J. L. (1977). Modification of the behavioral hierarchy of *Pleurobranchaea*. *J. Comp. Physiol.* 117 99–125. 10.1007/bf00605525

[B48] Davis W. J. Siegler M. V. S. Mpitsos G. J. (1973). Distributed neuronal oscillators and efference copy in the feeding system of *Pleurobranchaea*. *J. Neurophysiol.* 36 258–274. 10.1152/jn.1973.36.2.258 4350359

[B49] Denizot J. P. Clausse S. Elekes K. Geffard M. Grant K. Libouban S. (1987). Convergence of electrotonic club endings, GABA- and serotoninergic terminals on second order neurons of the electrosensory pathway in mormyrid fish, *Gnathonemus petersii* and *Brienomyrus niger* (Teleostei). *Cell Tissue Res.* 249 301–309. 10.1007/bf00215512 2441869

[B50] Doupe A. J. Kuhl P. K. (1999). Birdsong and human speech: common themes and mechanisms. *Annu. Rev. Neurosci.* 22 567–631. 10.1146/annurev.neuro.22.1.567 10202549

[B51] Duhamel J. R. Colby C. L. Goldberg M. E. (1992). The updating of the representation of visual space in parietal cortex by intended eye movements. *Science* 255 90–92. 10.1126/science.1553535 1553535

[B52] Easter S. S. Johns P. R. (1974). Horizontal compensatory eye movements in goldfish (*Carassius auratus*). *J. Comp. Physiol.* 92 37–57. 10.1007/bf00696525

[B53] Edwards D. Heitler W. Krasne F. (1999). Fifty years of a command neuron: the neurobiology of escape behavior in the crayfish. *Trends Neurosci.* 22 153–161. 10.1016/S0166-2236(98)01340-X 10203852

[B54] Elekes K. Szabo T. (1985). The mormyrid brainstem—III. Ultrastructure and synaptic organization of the medullary “pacemaker” nucleus. *Neuroscience* 15 431–443. 10.1016/0306-4522(85)90224-6 4022333

[B55] Eliades S. J. Wang X. (2019). Corollary discharge mechanisms during vocal production in marmoset monkeys. *Biol. Psychiatry Cogn. Neurosci. Neuroimaging* 4 805–812. 10.1016/j.bpsc.2019.06.008 31420219 PMC6733626

[B56] Enger P. S. Libouban S. Szabo T. (1976a). Fast conducting electrosensory pathway in the mormyrid fish, *Gnathonemus petersii*. *Neurosci. Lett.* 2 133–136. 10.1016/0304-3940(76)90004-5 19604830

[B57] Enger P. S. Libouban S. Szabo T. (1976b). Rhombo-mesencephalic connections in the fast conducting electrosensory system of the mormyrid fish, *Gnathonemus peter*sii. An HRP study. *Neurosci. Lett.* 3 239–243. 10.1016/0304-3940(76)90049-5 19604893

[B58] Ford J. M. Mathalon D. H. Heinks T. Kalba S. Faustman W. O. Roth W. T. (2001). Neurophysiological evidence of corollary discharge dysfunction in Schizophrenia. *Am. J. Psychiat.* 158 2069–2071. 10.1176/appi.ajp.158.12.2069 11729029

[B59] Friedman M. A. Hopkins C. D. (1998). Neural substrates for species recognition in the time-coding electrosensory pathway of mormyrid electric fish. *J. Neurosci.* 18 1171–1185. 10.1523/JNEUROSCI.18-03-01171.1998 9437037 PMC6792764

[B60] Fukutomi M. Carlson B. A. (2020). Signal diversification is associated with corollary discharge evolution in weakly electric fish. *J. Neurosci.* (in press). 10.1523/JNEUROSCI.0875-20.2020 32661026 PMC7424872

[B61] Gallant J. R. Traeger L. L. Volkening J. D. Moffett H. Chen P.-H. Novina C. D. (2014). Genomic basis for the convergent evolution of electric organs. *Science* 344 1522–1525. 10.1126/science.1254432 24970089 PMC5541775

[B62] Gallese V. Fadiga L. Fogassi L. Rizzolatti G. (1996). Action recognition in the premotor cortex. *Brain* 119 593–609. 10.1093/brain/119.2.593 8800951

[B63] Gillette R. Davis W. J. (1977). The role of the metacerebral giant neuron in the feeding behavior of *Pleurobranchaea*. *J Comp. Physiol.* 116 129–159. 10.1007/bf00605400

[B64] Goldberg M. E. Wurtz R. H. (1972). Activity of superior colliculus in behaving monkey. I. Visual receptive fields of single neurons. *J. Neurophysiol.* 35 542–559. 10.1152/jn.1972.35.4.542 4624739

[B65] Grant K. Bell C. C. Clausse S. Ravaille M. (1986). Morphology and physiology of the brainstem nuclei controlling the electric organ discharge in mormyrid fish. *J. Comp. Neurol.* 245 514–530. 10.1002/cne.902450407 3700711

[B66] Griffin D. R. (1958). *Listening in the Dark.* New Haven, CT: Yale University Press.

[B67] Harris A. J. (1965). Eye movements of the dogfish *Squalus acanthias* L. *J. Exp. Biol.* 43 107–138. 5884062 10.1242/jeb.43.1.107

[B68] Hedwig B. (2000b). Control of cricket stridulation by a command neuron: efficacy depends on the behavioral state. *J. Neurophysiol.* 83 712–722. 10.1152/jn.2000.83.2.712 10669487

[B69] Hedwig B. (2000a). A highly sensitive opto-electronic system for the measurement of movements. *J. Neurosci. Meth.* 100 165–171. 10.1016/s0165-0270(00)00255-7 11040380

[B70] Heiligenberg W. (1969). The effect of stimulus chirps on a cricket’s chirping (*Acheta domesticus*). *Z. Vergl. Physiologie* 65 70–97. 10.1007/bf00297990

[B71] Heiligenberg W. Kawasaki M. (1992). An internal current source yields immunity of electrosensory information processing to unusually strong jamming in electric fish. *J. Comp. Physiol. A* 171 309–316. 10.1007/bf00223961 1447722

[B72] Heiligenberg W. F. (1991). *Neural Nets in Electric Fish.* Cambridge, MA: MIT Press.

[B73] Hillman D. E. (1969). Light and electron microscopical study of the relationships between the cerebellum and the vestibular organ of the frog. *Exp. Brain Res.* 9 1–15. 10.1007/bf00235448 5808479

[B74] Hopkins C. D. (1986a). “Behavior of mormyridae,” in *Electroreception*, eds Bullock T. H. Heilgenberg W. (New York, NY: John Wiley & Sons), 527–576.

[B75] Hopkins C. D. (1986b). Temporal structure of non-propagated electric communication signals. *Brain Behav. Evol.* 28 43–59. 10.1159/000118691 3567540

[B76] Hopkins C. D. (1999). Design features for electric communication. *J. Exp. Biol.* 202 1217–1228. 10210663 10.1242/jeb.202.10.1217

[B77] Johnstone J. R. Mark R. F. (1969). Evidence for efference copy for eye movements in fish. *Comp. Biochem. Physiol.* 30 931–939. 10.1016/0010-406x(69)90048-6 5347607

[B78] Kalmijn A. J. (1971). The electric sense of sharks and rays. *J. Exp. Biol.* 55 371–383. 5114029 10.1242/jeb.55.2.371

[B79] Kalmijn A. J. (1974). “The detection of electric fields from inanimate and animate sources other than electric organs,” in *Electroreceptors and Other Specialized Receptors in Lower Vertrebrates. Handbook of Sensory Physiology*, ed. Fessard A. (Berlin: Springer), 147–200. 10.1007/978-3-642-65926-3_5

[B80] Kawasaki M. (1993). Independently evolved jamming avoidance responses employ identical computational algorithms: a behavioral study of the African electric fish, *Gymnarchus nilotic*us. *J. Comp. Physiol. A* 173 9–22. 10.1007/bf00209614 8366474

[B81] Kawasaki M. (1994). The African wave-type electric fish, *Gymnarchus niloticus*, lacks corollary discharge mechanisms for electrosensory gating. *J. Comp. Physiol. A* 174 133–144. 10.1007/bf00193781 8145186

[B82] Kawasaki M. Heiligenberg W. (1990). Different classes of glutamate receptors and GABA mediate distinct modulations of a neuronal oscillator, the medullary pacemaker of a gymnotiform electric fish. *J. Neurosci.* 10 3896–3904. 10.1523/jneurosci.10-12-03896.1990 1980133 PMC6570047

[B83] Keller C. H. Maler L. Heiligenberg W. (1990). Structural and functional organization of a diencephalic sensory-motor interface in the gymnotiform fish, *Eigenmannia*. *J. Comp. Neurol.* 293 347–376. 10.1002/cne.902930304 1691214

[B84] Kennedy A. Wayne G. Kaifosh P. Alviña K. Abbott L. Sawtell N. B. (2014). A temporal basis for predicting the sensory consequences of motor commands in an electric fish. *Nat. Neurosci.* 17 416–422. 10.1038/nn.3650 24531306 PMC4070001

[B85] Kennedy G. Heiligenberg W. (1994). Ultrastructural evidence of GABA-ergic inhibition and glutamatergic excitation in the pacemaker nucleus of the gymnotiform electric fish, *Hypopomus*. *J. Comp. Physiol. A* 174 267–280. 10.1007/bf00240210 7908694

[B86] Konishi M. (1965). The role of auditory feedback in the control of vocalization in the white-crowned sparrow. *Z. Tierpsychol.* 22 770–783. 5874921

[B87] Kothari N. B. Wohlgemuth M. J. Moss C. F. (2018). Dynamic representation of 3D auditory space in the midbrain of the free-flying echolocating bat. *eLife* 7:e29053. 10.7554/eLife.29053 29633711 PMC5896882

[B88] Kovac M. P. Davis W. J. (1977). Behavioral choice: neural mechanisms in *Pleurobranchaea*. *Science* 198 632–634. 10.1126/science.918659 918659

[B89] Kovac M. P. Davis W. J. (1980). Neural mechanism underlying behavioral choice in *Pleurobranchaea*. *J. Neurophysiol.* 43 469–487. 10.1152/jn.1980.43.2.469 7381529

[B90] Krasne F. B. Bryan J. S. (1973). Habituation: regulation through presynaptic inhibition. *Science* 182 590–592. 10.1126/science.182.4112.590 4795747

[B91] Lambert F. M. Combes D. Simmers J. Straka H. (2012). Gaze stabilization by efference copy signaling without sensory feedback during vertebrate locomotion. *Curr. Biol.* 22 1649–1658. 10.1016/j.cub.2012.07.019 22840517

[B92] Libouban S. Szabo T. (1977). An integration centre of the mormyrid fish brain: the *Auricula cerebelli*. An HRP study. *Neurosci. Lett.* 6 115–119. 10.1016/0304-3940(77)90005-2 19605039

[B93] Lynch J. C. Hoover J. E. Strick P. L. (1994). Input to the primate frontal eye field from the substantia nigra, superior colliculus, and dentate nucleus demonstrated by transneuronal transport. *Exp. Brain Res.* 100 181–186. 10.1007/bf00227293 7813649

[B94] Lyon E. P. (1900). Compensatory motions in fishes. *Am. J. Physiol.* 4 77–82. 10.1152/ajplegacy.1900.4.2.77

[B95] Lyons-Warren A. M. Kohashi T. Mennerick S. Carlson B. A. (2013). Detection of submillisecond spike timing differences based on delay-line anticoincidence detection. *J. Neurophysiol.* 110 2295–2311. 10.1152/jn.00444.2013 23966672 PMC3841875

[B96] Maler L. (1973). The posterior lateral line lobe of a mormyrid fish — a golgi study. *J. Comp. Neurol.* 152 281–298. 10.1002/cne.901520305 4130105

[B97] Mark R. F. Davidson T. M. (1966). Unit responses from commissural fibers of optic lobes of fish. *Science* 152 797–799. 10.1126/science.152.3723.797 17797463

[B98] Markram H. Gerstner W. Sjöström P. J. (2011). A history of spike-timing-dependent plasticity. *Front. Syn. Neurosci.* 3:4. 10.3389/fnsyn.2011.00004 22007168 PMC3187646

[B99] Marler P. (1964). “Inheritance and learning in the development of animal vocalization,” in *Acoustic Behavior of Animals*, ed. Busnel M. C. (Amsterdam: Elsevier), 228–243.

[B100] Martínez-Silva L. Manjarrez E. Gutiérrez-Ospina G. Quevedo J. N. (2014). Electrophysiological representation of scratching CPG activity in the cerebellum. *PLoS One* 9:e109936. 10.1371/journal.pone.0109936 25350378 PMC4211676

[B101] McCrea R. A. Gdowski G. T. Boyle R. Belton T. (1999). Firing behavior of vestibular neurons during active and passive head movements: vestibulo-spinal and other non-eye-movement related neurons. *J. Neurophysiol.* 82 416–428. 10.1152/jn.1999.82.1.416 10400968

[B102] Meek J. Nieuwenhuys R. (1998). “Holosteans and teleosts,” in *The Central Nervous System of Vertebrates*, eds Nieuwenhuys R. Donkelaar H. J. Nicholson C. (New York: Springer), 759–937. 10.1007/978-3-642-18262-4_15

[B103] Montgomery J. C. (1984). Noise cancellation in the electrosensory system of the thornback ray; common mode rejection of input produced by the animal’s own ventilatory movement. *J. Comp. Physiol. A* 155 103–111. 10.1007/bf00610935

[B104] Montgomery J. C. Bodznick D. (1994). An adaptive filter that cancels self-induced noise in the electrosensory and lateral line mechanosensory systems of fish. *Neurosci. Lett.* 174 145–148. 10.1016/0304-3940(94)90007-8 7970170

[B105] Morton S. M. Bastian A. J. (2004). Cerebellar control of balance and locomotion. *Neuroscientist* 10 247–259. 10.1177/1073858404263517 15155063

[B106] Mugnaini E. Maler L. (1987). Cytology and immunocytochemistry of the nucleus of the lateral line lobe in the electric fish *Gnathonemus petersii* (mormyridae): evidence suggesting that GABAergic synapses mediate an inhibitory corollary discharge. *Synapse* 1 32–56. 10.1002/syn.890010107 2850619

[B107] Müller-Preuss P. Newman J. D. Jürgens U. (1980). Anatomical and physiological evidence for a relationship between the ‘cingular’ vocalization area and the auditory cortex in the squirrel monkey. *Brain Res.* 202 307–315. 10.1016/0006-8993(80)90143-2 7437905

[B108] Müller-Preuss P. Ploog D. (1981). Inhibition of auditory cortical neurons during phonation. *Brain Res.* 215 61–76. 10.1016/0006-8993(81)90491-1 7260601

[B109] Murphey R. K. Palka J. (1974). Efferent control of cricket giant fibres. *Nature* 248 249–251. 10.1038/248249a0

[B110] Nachtigal P. E. Schuller G. (2014). “Hearing during echolocation in whales and bats,” in *Biosonar*, eds Surlykke A. Nachtigal P. E. Fay R. R. Popper A. N. (New York, NY: Springer), 143–168. 10.1007/978-1-4614-9146-0_5

[B111] Neuweiler G. (2003). Evolutionary aspects of bat echolocation. *J. Comp. Physiol. A* 189 245–256. 10.1007/s00359-003-0406-2 12743729

[B112] New J. G. Bodznick D. (1990). Medullary electrosensory processing in the little skate. *J. Comp. Physiol. A* 167 295–307. 10.1007/bf00188121 2213659

[B113] Nocke H. (1972). Physiological aspects of sound communication in crickets (*Gryllus campestris* L.). *J. Comp. Physiol.* 80 141–162. 10.1007/bf00696487

[B114] Nottebohm F. (2005). The neural basis of birdsong. *PLoS Biol.* 3:e164. 10.1371/journal.pbio.0030164 15884976 PMC1110917

[B115] Oertel D. Young E. D. (2004). What’s a cerebellar circuit doing in the auditory system? *Trends Neurosci.* 27 104–110. 10.1016/j.tins.2003.12.001 15102490

[B116] Paul D. H. Roberts B. L. (1977). Studies on a primitive cerebellar cortex I. The anatomy of the lateral-line lobes of the dogfish, *Scyliorhinus canicula*. *Proc. R. Soc. B* 195 453–466. 10.1098/rspb.1977.0020 15265

[B117] Perks K. Sawtell N. B. (2019). “Influences of motor systems on electrosensory processing,” in *Electroreception: Fundamental Insights form Comparative Approaches*, eds Carlson B. A. Sisneros J. A. Popper A. N. Fay R. R. (New York, NY: Springer), 315–338. 10.1007/978-3-030-29105-1_11

[B118] Pieper F. Jürgens U. (2003). Neuronal activity in the inferior colliculus and bordering structures during vocalization in the squirrel monkey. *Brain Res.* 979 153–164. 10.1016/s0006-8993(03)02897-x 12850582

[B119] Poulet J. F. Hedwig B. (2003a). A corollary discharge mechanism modulates central auditory processing in singing crickets. *J. Neurophysiol.* 89 1528–1540. 10.1152/jn.0846.2002 12626626

[B120] Poulet J. F. Hedwig B. (2003b). Corollary discharge inhibition of ascending auditory neurons in the stridulating cricket. *J. Neurosci.* 23 4717–4725. 10.1523/JNEUROSCI.23-11-04717.2003 12805311 PMC6740803

[B121] Poulet J. F. Hedwig B. (2006). The cellular basis of a corollary discharge. *Science* 311 518–522. 10.1126/science.1120847 16439660

[B122] Poulet J. F. Hedwig B. (2007). New insights into corollary discharges mediated by identified neural pathways. *Trends Neurosci.* 30 14–21. 10.1016/j.tins.2006.11.005 17137642

[B123] Poulet J. F. A. Hedwig B. (2002). A corollary discharge maintains auditory sensitivity during sound production. *Nature* 418 872–876. 10.1038/nature00919 12192409

[B124] Prather J. F. Peters S. Nowicki S. Mooney R. (2008). Precise auditory–vocal mirroring in neurons for learned vocal communication. *Nature* 451 305–310. 10.1038/nature06492 18202651

[B125] Requarth T. Sawtell N. B. (2011). Neural mechanisms for filtering self-generated sensory signals in cerebellum-like circuits. *Curr. Opin. Neurobiol.* 21 602–608. 10.1016/j.conb.2011.05.031 21704507

[B126] Roberts B. L. Russell I. J. (1972). The activity of lateral-line efferent neurones in stationary and swimming dogfish. *J. Exp. Biol.* 57 435–448. 4634495 10.1242/jeb.57.2.435

[B127] Roberts T. F. Hisey E. Tanaka M. Kearney M. G. Chattree G. Yang C. F. (2017). Identification of a motor-to-auditory pathway important for vocal learning. *Nat. Neurosci.* 20 978–986. 10.1038/nn.4563 28504672 PMC5572074

[B128] Roy J. E. Cullen K. E. (2001). Selective processing of vestibular reafference during self-generated head motion. *J. Neurosci.* 21 2131–2142. 10.1523/jneurosci.21-06-02131.2001 11245697 PMC6762599

[B129] Roy J. E. Cullen K. E. (2004). Dissociating self-generated from passively applied head motion: neural mechanisms in the vestibular nuclei. *J. Neurosci.* 24 2102–2111. 10.1523/jneurosci.3988-03.2004 14999061 PMC6730417

[B130] Sawtell N. B. Williams A. Bell C. C. (2005). From sparks to spikes: information processing in the electrosensory systems of fish. *Curr. Opin. Neurobiol.* 15 437–443. 10.1016/j.conb.2005.06.006 16009545

[B131] Schneider D. M. Mooney R. (2018). How movement modulates hearing. *Annu. Rev. Neurosci.* 41 553–572. 10.1146/annurev-neuro-072116-031215 29986164 PMC6201761

[B132] Schuller G. (1979). Vocalization influences auditory processing in collicular neurons of the CF-FM-bat, *Rhinolophus ferrumequinum*. *J. Comp. Physiol.* 132 39–46. 10.1007/bf00617730

[B133] Siegler M. V. Mpitsos G. J. Davis W. J. (1974). Motor organization and generation of rhythmic feeding output in buccal ganglion of *Pleurobranchaea*. *J. Neurophysiol.* 37 1173–1196. 10.1152/jn.1974.37.6.1173 4373547

[B134] Singla S. Dempsey C. Warren R. Enikolopov A. G. Sawtell N. B. (2017). A cerebellum-like circuit in the auditory system cancels responses to self-generated sounds. *Nat. Neurosci.* 20 943–950. 10.1038/nn.4567 28530663 PMC5525154

[B135] Sommer M. A. Wurtz R. H. (2002). A pathway in primate brain for internal monitoring of movements. *Science* 296 1480–1482. 10.1126/science.1069590 12029137

[B136] Sommer M. A. Wurtz R. H. (2004). What the brain stem tells the frontal cortex. II. Role of the SC-MD-FEF pathway in corollary discharge. *J. Neurophysiol.* 91 1403–1423. 10.1152/jn.00740.2003 14573557

[B137] Sommer M. A. Wurtz R. H. (2006). Influence of the thalamus on spatial visual processing in frontal cortex. *Nature* 444 374–377. 10.1038/nature05279 17093408

[B138] Sparks D. L. Hartwich-Young R. (1989). The deep layers of the superior colliculus. *Rev. Oculomot. Res.* 3 213–255. 2486324

[B139] Sperry R. W. (1950). Neural basis of the spontaneous optokinetic response produced by visual inversion. *J. Comp. Physiol. Psychol.* 43 482–489. 10.1037/h0055479 14794830

[B140] Stehouwer D. J. (1987). Compensatory eye movements produced during fictive swimming of a deafferented, reduced preparation in vitro. *Brain Res.* 410 264–268. 10.1016/0006-8993(87)90323-4 3496141

[B141] Straka H. Simmers J. Chagnaud B. P. (2018). A new perspective on predictive motor signaling. *Curr. Biol.* 28 R232–R243. 10.1016/j.cub.2018.01.033 29510116

[B142] Suga N. Schlegel P. (1972). Neural attenuation of responses to emitted sounds in echolocating bats. *Science* 177 82–84. 10.1126/science.177.4043.82 4557490

[B143] Suga N. Shimozawa T. (1974). Site of neural attenuation of responses to self-vocalized sounds in echolocating bats. *Science* 183 1211–1213. 10.1126/science.183.4130.1211 4812353

[B144] Troyer T. W. Doupe A. J. (2000a). An associational model of birdsong sensorimotor learning I. Efference copy and the learning of song syllables. *J. Neurophysiol.* 84 1204–1223. 10.1152/jn.2000.84.3.1204 10979996

[B145] Troyer T. W. Doupe A. J. (2000b). An associational model of birdsong sensorimotor learning II. Temporal hierarchies and the learning of song sequence. *J. Neurophysiol.* 84 1224–1239. 10.1152/jn.2000.84.3.1224 10979997

[B146] Tzounopoulos T. Kim Y. Oertel D. Trussell L. O. (2004). Cell-specific, spike timing–dependent plasticities in the dorsal cochlear nucleus. *Nat. Neurosci.* 7 719–725. 10.1038/nn1272 15208632

[B147] Tzounopoulos T. Rubio M. E. Keen J. E. Trussell L. O. (2007). Coactivation of pre- and postsynaptic signaling mechanisms determines cell-specific spike-timing-dependent plasticity. *Neuron* 54 291–301. 10.1016/j.neuron.2007.03.026 17442249 PMC2151977

[B148] Umeno M. M. Goldberg M. E. (1997). Spatial processing in the monkey frontal eye field. I. predictive visual responses. *J. Neurophysiol.* 78 1373–1383. 10.1152/jn.1997.78.3.1373 9310428

[B149] Vélez A. Carlson B. A. (2016). Detection of transient synchrony across oscillating receptors by the central electrosensory system of mormyrid fish. *eLife* 5:e16851. 10.7554/eLife.16851 27328322 PMC4954753

[B150] von der Emde G. (1999). Active electrolocation of objects in weakly electric fish. *J. Exp. Biol.* 202 1205–1215. 10210662 10.1242/jeb.202.10.1205

[B151] von der Emde G. Bell C. C. (2003). ““Active electrolocation and its neural processing in mormyrid,”,” in *Sensory Processing in Aquatic Environments*, eds Collin S. P. Marshall J. (New York, NY: Springer), 92–107. 10.1007/978-0-387-22628-6_5

[B152] von der Emde G. Sena L. G. Niso R. Grant K. (2000). The midbrain precommand nucleus of the mormyrid electromotor network. *J. Neurosci.* 20 5483–5495. 10.1523/jneurosci.20-14-05483.2000 10884332 PMC6772327

[B153] von Holst E. Mittelstaedt H. (1950). Das reafferenzprinzip. *Naturwissenschaften* 37 464–476. 10.1007/bf00622503

[B154] Weeg M. S. Land B. R. Bass A. H. (2005). Vocal pathways modulate efferent neurons to the inner ear and lateral line. *J. Neurosci.* 25 5967–5974. 10.1523/jneurosci.0019-05.2005 15976085 PMC6724790

[B155] Wenzel B. Hedwig B. (1999). Neurochemical control of cricket stridulation revealed by pharmacological microinjections into the brain. *J. Exp. Biol.* 202 2203–2216. 10409491 10.1242/jeb.202.16.2203

[B156] Wiersma C. A. Yamaguchi T. (1967). Integration of visual stimuli by the crayfish central nervous system. *J. Exp. Biol.* 47 409–431. 5592409 10.1242/jeb.47.3.409

[B157] Wiersma C. A. G. (1947). Giant nerve fiber system of the crayfish. A contribution to comparative physiology of synapse. *J. Neurophysiol.* 10 23–38. 10.1152/jn.1947.10.1.23 20279137

[B158] Wullimann M. F. Northcutt R. G. (1990). Visual and electrosensory circuits of the diencephalon in mormyrids: an evolutionary perspective. *J. Comp. Neurol.* 297 537–552. 10.1002/cne.902970407 2384612

[B159] Xu-Friedman M. Hopkins C. (1999). Central mechanisms of temporal analysis in the knollenorgan pathway of mormyrid electric fish. *J. Exp. Biol.* 202 1311–1318. 10210671 10.1242/jeb.202.10.1311

[B160] Yamada J. Noda H. (1987). Afferent and efferent connections of the oculomotor cerebellar vermis in the macaque monkey. *J. Comp. Neurol.* 265 224–241. 10.1002/cne.902650207 3320110

[B161] Yartsev M. M. Ulanovsky N. (2013). Representation of three-dimensional space in the hippocampus of flying bats. *Science* 340 367–372. 10.1126/science.1235338 23599496

[B162] Zipser B. Bennett M. V. (1976). Interaction of electrosensory and electromotor signals in lateral line lobe of a mormyrid fish. *J. Neurophysiol.* 39 713–721. 10.1152/jn.1976.39.4.713 184257

